# Qinggan Jianpi formula attenuates atherosclerosis by suppressing macrophage lactate transport to activate repair genes via H3K18 lactylation

**DOI:** 10.1186/s13020-026-01394-0

**Published:** 2026-04-24

**Authors:** Xuefeng Zhuang, Rui Zhang, Jiatong Sun, Wenli Song, Jing Wang, Yvna Han, Jinji Wang, Hongzhu Chen, Zhijie Zhu, Weijia Liu, Lijing Li

**Affiliations:** 1 Department of Pharmacology, School of Pharmaceutical Sciences, Changchun University of Chinese Medicine, Changchun, Jilin, 130117 China; 2 Tonghua Golden-Horse Pharmaceutical Industry Co., Ltd, Tonghua, Jilin, 134000 China; 3 Jilin University, Changchun, Jilin, 130117 China

**Keywords:** H3K18la, Lactate, Macrophage, Metabolic reprogramming, Atherosclerosis

## Abstract

**Background:**

Atherosclerosis (AS) is a complex vascular disease characterized by lipid accumulation, chronic inflammation, and immune dysregulation. Qinggan Jianpi Formula (QGJP), a traditional Chinese medicinal preparation, is widely used for treating lipid metabolism disorders. However, the mechanisms of action and active components remain unclear. These uncertainties restrict its clinical use and necessitate systematic research to clarify them. This study aims to investigate the therapeutic effects of QGJP on AS and to elucidate the role of suppressing macrophage M1 polarization in this process, mediated by the regulation of lactate transport and the promotion of histone lactylation.

**Methods:**

In this study, first, major chemical components of QGJP were identified via UHPLC-HRMS. We employed a high-fat diet (HFD) fed ApoE^⁻/⁻^ C57BL/6 mice to establish an AS model, along with in vitro models of ox-LDL-induced lipid injury in HUVECs and ox-LDL-induced foam cell formation in THP-1, followed by QGJP treatment. We elucidated the therapeutic effects and underlying mechanisms of QGJP in AS through multiple approaches, including protecting endothelial cells and regulating macrophage polarization.

**Results:**

QGJP significantly reduced blood lipids, modulated plaque lipid and collagen content, and alleviated aortic pathological damage in AS mice. Moreover, QGJP downregulated the expression of matrix metalloproteinases, adhesion molecules, and chemokines, enhanced endothelial migration capacity, and inhibited monocyte-endothelial adhesion. Further analysis of macrophage polarization phenotypes revealed that QGJP significantly modulated their polarization state. Mechanistically, QGJP suppressed the expression of key glycolytic enzymes while promoting that of FH. Consequently, it reversed the increase in glycolytic activity observed in macrophages during atherosclerosis. Furthermore, QGJP regulated lactate transport by suppressing MCT4 expression. This modulation orchestrated histone H3K18 lactylation, which in turn activated repair-related gene programs in macrophages. Through UHPLC-HRMS analysis, 47 bioactive constituents of QGJP were identified. Experimental validation confirmed that SAA, LA, and CAB effectively inhibited M1 macrophage polarization and activated the expression of proteins related to reparative genes.

**Conclusions:**

This study establishes the critical role of metabolic reprogramming and epigenetic regulation in AS progression. Our findings suggest that a mechanism whereby QGJP alleviates AS may involve the inhibition of the HIF-1α/MCT4 axis and lactate transport, which regulates histone H3K18 lactylation to promote a reparative macrophage phenotype.

**Supplementary Information:**

The online version contains supplementary material available at 10.1186/s13020-026-01394-0.

## Introduction

Atherosclerosis (AS) is a complex vascular disease characterized by lipid deposition, chronic inflammation, and immune dysregulation [1]. AS and its related cardio-cerebrovascular complications, such as acute myocardial infarction and ischemic stroke, are leading causes of death globally among non-communicable diseases.

The progression of AS plaques disrupts blood flow to the heart and brain, leading to severe clinical symptoms. The primary goals of AS treatment are to delay or stabilize plaque progression and prevent serious cardiovascular events such as myocardial infarction and stroke [2]. Current pharmacological strategies focus primarily on lipid-lowering therapy, with reduction of low-density lipoprotein cholesterol as the cornerstone [3]. When medication fails to control symptoms or when vascular stenosis is extremely severe, surgical “clearing” of the vessels is required [4]. However, restenosis and lumen thrombosis following these procedures remain major clinical challenges in treating AS. Therefore, innovative approaches are urgently needed to manage AS and mitigate post-injury restenosis and thrombotic events.

Current research indicates that AS is not merely simple vascular calcification but a complex chronic pathological process involving multiple factors such as inflammatory responses and metabolic disorders. The initiation of AS involves widening gaps between arterial endothelial cells, allowing LDL deposition onto the vascular endothelium. This process enhances endothelial cell adhesiveness, attracting monocytes and T lymphocytes from the bloodstream to adhere and guiding leukocytes into the subendothelial space [5]. LDL deposited beneath the endothelium undergoes oxidative modification by local free radicals, transforming into oxidized LDL (ox-LDL). Monocytes migrate to the subendothelial space and differentiate into macrophages, which extensively phagocytose ox-LDL via scavenger receptors. This leads to cytoplasmic filling with lipid droplets, forming foam cells. Aggregation of numerous foam cells creates macroscopically visible lipid streaks, representing the earliest pathological lesion of AS [6].

Monocytes are key drivers of AS plaque formation and progression. Under pathological conditions, they adhere to dysfunctional endothelial cells and differentiate into macrophages. As highly plastic immune cells, their polarization states play critical roles in AS [7]. Recent studies indicate that macrophage metabolic reprogramming is closely linked to their polarization phenotype: M1 macrophages rely on glycolysis for rapid energy production, whereas M2 macrophages prefer to maintain homeostasis through oxidative phosphorylation (OXPHOS) and the tricarboxylic acid (TCA) cycle. Metabolite-regulated post-translational modifications (PTMs), such as lactylation, acetylation, and succinylation, form a key regulatory hub that dynamically mediates the impact of metabolic state on immune phenotype, thereby linking metabolic reprogramming to macrophage function. Specific metabolites can precisely target particular amino acid residues on histones, thereby regulating downstream signaling pathways or gene expression programs and modulating cell function [8]. Inflammatory macrophages, highly dependent on glycolysis, produce large amounts of lactate, which serves as a direct substrate for histone lactylation. This modification translates the high-glycolytic metabolic state of inflammatory macrophages into a functionally specific gene expression program, driving a functional shift from a pro-inflammatory towards a tissue-reparative phenotype and facilitating inflammation resolution and tissue homeostasis [9]. In macrophages, lactate serves as a key metabolic product whose concentration fluctuations play a crucial role in functional transitions. Monocarboxylate transporter 4 (MCT4) is the primary lactate transporter on the surface of glycolytic cells, with its core function being the outward efflux of intracellular lactate. This process limits intracellular lactate accumulation, thereby maintaining glycolytic metabolic homeostasis [10]. Studies have confirmed that abnormal overexpression of MCT4 is closely associated with tumorigenesis and progression. In malignant tumors such as breast cancer, hepatocellular carcinoma, and lymphoma, MCT4 enhances lactate efflux, thereby supporting the Warburg effect in tumor cells and promoting tumor proliferation and invasion [11, 12]. Notably, as core glycolytic cells within the inflammatory microenvironment, the phenotypic conversion of macrophages may also be closely linked to MCT4-mediated lactate transport functions. In recent years, research on AS has evolved from an initial focus on macrophage metabolic disorders to exploration of metabolism-driven epigenetic remodeling mechanisms. Metabolites influence macrophage gene expression programs through regulating epigenetic modifications, ultimately contributing to the pathological progression of AS [13]. Previous studies have shown that inhibiting MCT4 expression promotes intracellular lactate accumulation. High lactate concentrations act as an epigenetic substrate, facilitating lactate-mediated modifications that enhance the expression of macrophage repair-related genes and alter macrophage function, thereby exerting anti-AS effects [14]. Therefore, targeting the molecular mechanism by which MCT4 links macrophage metabolic reprogramming to epigenetic regulation represents a promising therapeutic strategy against AS.

Traditional Chinese medicine (TCM) is widely used worldwide for treating chronic inflammatory diseases due to its long-term safety profile. Qinggan Jianpi Formula (QGJP), a traditional Chinese herbal compound comprising 11 herbs including *Crataegus pinnatifida* Bunge, *Alisma plantago-aquatica* L., *Artemisia capillaris* Thunb., *Salvia miltiorrhiza* Bunge, *Astragalus membranaceus* (Fisch.) Bunge, *Rheum palmatum* L., *Pleuropterus multiflorus* (Thunb.) Turcz. ex Nakai, *Plantago asiatica* L., *Polygonum cuspidatum* Sieb. et Zucc., *Bupleurum chinense* DC.*,* and *Senna tora* (L) Roxb*.* In TCM theory, QGJP is designed to fortify the spleen and resolve dampness, as well as clear the liver and remove lipids. AS, categorized under TCM concepts such as “meridian accumulation” and “phlegm-turbidity” has a core pathogenesis closely associated with “spleen dysfunction leading to dampness retention, liver constraint causing lipid stagnation.” [15]. Our previous research demonstrated QGJP’s efficacy in regulating lipid metabolism and inhibiting inflammation. Furthermore, studies have shown that active constituents of QGJP, such as tanshinone IIA, hawthorn flavonoids, and astragalosides, exhibit significant anti-AS effects [16–18].

However, the precise role and underlying mechanisms of the integrated QGJP in treating AS remain unclear. To investigate this, we employed HFD fed ApoE^⁻/⁻^ mice to establish an AS model and comprehensively evaluated the therapeutic effect of QGJP, focusing on endothelial protection and macrophage polarization. Our results demonstrated that QGJP ameliorated endothelial function and suppressed pro-inflammatory M1 macrophage polarization. Mechanistically, we identified a crucial role for HIF-1α/MCT4-mediated lactate metabolism in the immunometabolic regulation of AS. QGJP and its active components inhibited the HIF-1α/MCT4 axis, leading to intracellular lactate accumulation in macrophages. This subsequently induced histone H3K18 lactylation, activated a reparative gene expression program, promoted an M2-like functional shift, and ultimately resulted in plaque microenvironment remodeling and disease attenuation. This work not only reveals a novel therapeutic strategy targeting the lactate metabolism-epigenetics cross-talk for AS but also provides a theoretical foundation for developing immunotherapeutic strategies based on metabolic reprogramming regulation.

## Materials and methods

### Animal experiment

A total of 30 mice were used in this study: 6 C57BL/6 mice served as the Control group, and 24 ApoE^⁻/⁻^ C57BL/6 mice were randomly divided by weight into four groups: the model group, the QGJP high-dose group (QGJP-H, 3.6 g/kg/d), the QGJP low-dose group (QGJP-L, 1.8 g/kg/d), and the atorvastatin control group (Ator, 3.0 mg/kg/d). The clinical dose of Qinggan Jianpi formula was converted to a mouse-equivalent dose to establish the low-dose treatment group (QGJP-L). A high-dose group (QGJP-H) was administered at twice the dosage of the QGJP-L group. All mice were supplied by Beijing Vital River Laboratory Animal Technology Co., Ltd. (License No.: SCXK (Jing) 2102–0001). The mice were housed in the specific pathogen-free animal facility of the University (SCXK (Ji) 2023–0015) under a 12-h light/dark cycle, controlled temperature (23–25 °C), and humidity (40–70%). All experimental procedures were performed in accordance with institutional guidelines and approved by the Animal Ethics Committee of Changchun University of Traditional Chinese Medicine (approval number: 2023–672). Following a one-week acclimatization period. For the first 12 weeks, all groups except the control group were fed a HFD (D12108C, Research Diets, USA) to induce the AS model. Subsequently, over the following 8 weeks, the mice received daily oral gavage according to their group assignments: the QGJP-H, QGJP-L, and Ator groups received their respective treatments, while the Control and Model groups received an equivalent volume of distilled water (Fig. 12a). At the end of the experimental period, cardiac function (ejection fraction and fractional shortening), cerebral blood flow, and serum lipid levels were measured.

### Preparation of QGJP drug-containing serum

Adult male Sprague-Dawley rats weighing 250 ± 20 g were randomly divided into drug administration and control groups. Rats in the drug-treated group received QGJP by oral gavage at twice the clinical equivalent dose for 7 consecutive days. Within 30 min after the last administration, the rats were anesthetized with sodium pentobarbital (86-01-22, Chemical Reagent Procurement and Supply Station Subpackaging Plant, China), and blood was collected from the abdominal aorta. The blood samples were centrifuged at 3000 rpm for 15 min to obtain serum. The serum was then inactivated by heat treatment at 56 °C for 30 min, sterilized by filtration through a 0.22 μm membrane, aliquoted, and stored at −80 °C for subsequent experiments.

### Preparation and UHPLC-HRMS analysis of QGJP

QGJP is composed of eleven herbal medicines: *Crataegus pinnatifida* Bunge, *Alisma plantago-aquatica* L., *Artemisia capillaris* Thunb., *Salvia miltiorrhiza* Bunge, *Astragalus membranaceus* (Fisch.) Bunge, *Rheum palmatum* L., *Pleuropterus multiflorus* (Thunb.) Turcz. ex Nakai, *Plantago asiatica* L., *Polygonum cuspidatum* Sieb. et Zucc., *Bupleurum chinense* DC.*,* and *Senna tora* (L) Roxb*.* All herbs were provided and authenticated by TongRenTang Chinese Medicine (Beijing, China). All materials met the quality standards stipulated by the Chinese Pharmacopoeia (2020 edition), and voucher specimens have been deposited in our laboratory for future reference.

For extraction, *Crataegus pinnatifida* Bunge and *Salvia miltiorrhiza* Bunge were refluxed three times with 50% ethanol. After filtration, ethanol was recovered and the extract was concentrated. The herbal residue was then decocted three times with the remaining nine herbs in water. The aqueous extract was filtered, concentrated, and combined with the ethanol extract. Subsequently, three volumes of ethanol were added to the mixture under continuous stirring. After standing for 12 h, the solution was filtered, ethanol was recovered, and the final extract was concentrated to a density of 0.388 g/mL.

The major absorbed bioactive components of QGJP were characterized using UHPLC-HRMS. Chromatographic separation was performed on a Vanquish UHPLC system (Thermo Scientific, USA) equipped with an HSS-T3 column (100 × 2.1 mm, 1.8 μm; Waters) maintained at 35 °C. The mobile phases consisted of (A) water with 0.1% formic acid and (B) acetonitrile with 0.1% formic acid (LC–MS grade). A gradient elution was applied at a flow rate of 0.3 mL/min as follows: 5% B for 1 min, increased to 98% B over 16 min, returned to 5% B in 0.5 min, and held for 2.5 min. Mass spectrometric detection was conducted on a Q-Exactive HFX instrument (Thermo Fisher Scientific, Germany) operated in both positive and negative electrospray ionization (ESI) modes with data-dependent acquisition (DDA). Full MS scans covered *m/z* 90–1300, and the top 10 most intense ions were selected for MS/MS analysis using stepped HCD collision energies of 20, 40, and 60 eV. The capillary and probe heater temperatures were set to 320 °C and 350 °C, respectively.

The three-dimensional structure of the target protein was retrieved from the RCSB Protein Data Bank (http://www.rcsb.org/pdb/) in PDB format (PDB ID4H6J) and preprocessed using PyMOL software, including the removal of water molecules and ions. Subsequently, the protein structure was optimized in MOE by adding hydrogen atoms and calculating partial charges, followed by export in PDBQT format. Ligand structures were obtained from the PubChem database (https://pubchem.ncbi.nlm.nih.gov/) in SDF format, energy-minimized, and converted to MOL files. Molecular docking was performed using MOE, and the docking results were evaluated primarily based on binding affinity and the number of hydrogen bonds between the compounds and the target protein. Lower binding energy and a greater number of hydrogen bonds indicate more stable binding and a higher likelihood of effective target-ligand interaction. Finally, PyMOL was utilized to generate 3D and 2D interaction diagrams, providing visual representation of binding site interactions.

### Cell culture

Human Umbilical Vein Endothelial Cells (HUVECs) were obtained from the Cell Bank of Type Culture Collection (Chinese Academy of Sciences, Shanghai). HUVECs was cultured in ECM medium (ScienCell Research Laboratories, USA) supplemented with 10% fetal bovine serum (ScienCell Research Laboratories, USA) and 5% penicillin-streptomycin (ScienCell Research Laboratories, USA). HUVECs were induced by 200 µg/mL oxidized low-density lipoprotein (ox-LDL, Yiyuan biotechnologies, China) for 24 h. At the time of modeling, the cells received their respective drug treatments according to the group assignment, and the treatment lasted 24 h.

The human monocytic cell line (THP-1) was obtained from the National Collection of Authenticated Cell Cultures and cultured in RPMI 1640 medium (Thermo Fisher Scientific, USA) supplemented with 10% fetal bovine serum (Thermo Fisher Scientific, USA) and 5% penicillin-streptomycin (Solarbio Life Science, China). THP-1 cells were induced by 100 ng/mL phorbol 12-myristate 13-acetate (PMA, Sigma-Aldrich, USA) for 24 h to develop into adherent macrophages and then exposed to 100 µg/mL oxidized low-density lipoprotein (ox-LDL, Yiyuan biotechnologies, China) for 24 h. At the time of modeling, the cells received their respective drug treatments according to the group assignment, and the treatment lasted 24 h. The working concentration of dimethyloxalylglycine (DMOG; Selleck Chemicals, Houston, TX, USA), an activator of hypoxia-inducible factor-1α (HIF-1α), is 20 μM, and it is prepared in culture medium. Cells were treated for 24 h with Salvianolic acid A (100 μM), Lithospermic acid (50 μM), and Calceolarioside B (25 μM). All compounds were obtained from Yuanye Bio-Technology (Shanghai, China) with purities > 98%.

### Aorta pathology analysis

The mouse aorta was dissected and fixed in 4% paraformaldehyde for 24 h. Sequential 5 μm-thick sections of the aorta were prepared for histological staining. The sections were subjected to hematoxylin and eosin (H&E) staining for general pathological observation and Sirius Red staining for evaluation of collagen deposition. For lipid detection, additional aortic samples were snap-frozen in liquid nitrogen. Cryosections were prepared at 6 μm thickness and stained with Oil Red O to visualize lipid droplet accumulation.

### Western blot

Cells and tissues were lysed on ice and centrifuged at 12,000 rpm for 20 min. Protein concentrations were measured by a BCA kit (P0010, Beyotime Biotechnology, China), and samples were separated by electrophoresis, transferred to PVDF membranes, and blocked with 5% skim milk. After incubation with primary and secondary antibodies, protein bands were detected using ECL substrate and quantified via ImageJ. The antibodies used in this study are listed below: MMP2 Monoclonal antibody (66,366–1-Ig, Proteintech, China), MMP9 Polyclonal antibody (83,768–3-RR, Proteintech, China), ICAM-1 Monoclonal antibody (60,299–1-Ig, Proteintech, China), MCP-1 Polyclonal antibody (26,161–1-AP, Proteintech, China), CD86 Monoclonal antibody (68,674–2-Ig, Proteintech, China), CD206 Polyclonal antibody (18,704–1-AP, Proteintech, China), IL-1β (16,806–1-AP, Proteintech, China), IL-6 Polyclonal antibody (26,404–1-AP, Proteintech, China), IL-10 Monoclonal antibody (60,269–1-Ig, Proteintech, China), HK2 Polyclonal antibody (22,029–1-AP, Proteintech, China), PFKFB3 Polyclonal antibody (13,763–1-AP, Proteintech, China), LDHA Recombinant monoclonal antibody (84,198–2-RR, Proteintech, China), FH Polyclonal antibody (11,375–1-AP, Proteintech, China), HIF-1α Polyclonal antibody (20,960–1-AP, Proteintech, China), MCT4 Polyclonal antibody (22,787–1-AP, Proteintech, China), P300 Polyclonal antibody (20,695–1-AP, Proteintech, China), H3K18la (PTM-1406RM, Jingjie Biotechnology, China), H3 Polyclonal antibody (17,168–1-AP, Proteintech, China), Anti-L-Lactyl Lysine Rabbit pAb (PTM-1401, Jingjie Biotechnology, China).

### IHC and IF staining

IF staining: THP-1 cells and paraffin-embedded aortic tissue sections were incubated with primary antibodies against CD86, CD206, F4/80, and CD31 overnight at 4 ℃, followed by 1-h incubation with fluorescent secondary antibodies in the dark. Sections were counterstained with DAPI, and images were captured using a fluorescence microscope (Leica DMi8, Solms, Germany). ImageJ software was used for image merging and analysis.

IHC staining: Tissue sections were deparaffinized, rehydrated through an ethanol gradient, and subjected to antigen retrieval using citrate buffer. Primary antibodies targeting ICAM-1 and MMP2 were applied overnight at 4 ℃. After washing, sections were incubated with secondary antibodies. Images were acquired using microscope (IX83, Olympus Corporation, Japan) and analyzed with ImageJ software.

### Scratch wound healing assay

HUVECs were seeded in 6 well plates. After cell attachment, they were treated with QGJP-containing serum and ox-LDL for 24 h, while the model and normal control groups received an equal volume of culture medium. Following treatment, the culture supernatant was removed, and a sterile 20 μL pipette tip was used to create a straight scratch in the confluent cell monolayer. The initial wound area was photographed under a microscope at marked locations. All groups were then incubated with medium. After 24 h, images were captured at the same pre-marked locations. The wound area was measured using ImageJ software, and cell migration ability was evaluated by comparing the changes in wound closure between groups.

### Cell adhesion assay

HUVECs were seeded in 6-well plates and treated with QGJP-containing serum and ox-LDL. THP-1 cells were adjusted to a density of 5 × 10^5^ cells/mL and labeled with CM-Dil (2.5 mg/mL, Beyotime Biotech, China) at 37 °C for 20 min. The labeled THP-1 cells were then added to the HUVECs monolayers and co-cultured for 1 h. After incubation, non-adherent THP-1 cells were removed by washing three times with sterile PBS. The number of adherent THP-1 cells was quantified under a fluorescence microscope.

### Measurement of intracellular ROS

Cells were seeded in 12 well plates and treated with QGJP-containing serum and ox-LDL. After treatment, the culture medium was removed and the cells were washed three times with PBS. Then, 300 μL of DCFH-DA working solution (S1105M, Beyotime Biotechnology, China) was added to each well, followed by incubation at 37 °C for 20 min. After removal of the staining solution, the cells were washed again with PBS. Finally, 300 μL of PBS was added to each well, and intracellular ROS levels were detected and imaged using fluorescence microscopy.

### Statistical analyses

Values are presented as the mean ± standard deviation (SD). The Shapiro-Wilk test was used to verify normality, and Levene’s test assessed the homogeneity of variance. The data met the criteria for normality and homogeneity of variance. Differences between groups were examined using one-way analysis of variance, with subsequent analysis using Tukey’s multiple comparisons test. Pearson correlation analysis was applied to data with homogeneity of variance, while Spearman correlation analysis was used for data that did not meet this assumption. SPSS version 26.0 (SPSS, Chicago, IL, USA) and GraphPad Prism 9.0 (Avenida, CA, USA) were used for preparing figures, respec­tively. *p*-value < 0.05 indicated statistically significant differences.

## Results

### QGJP inhibits AS progression

**Fig. 1:**
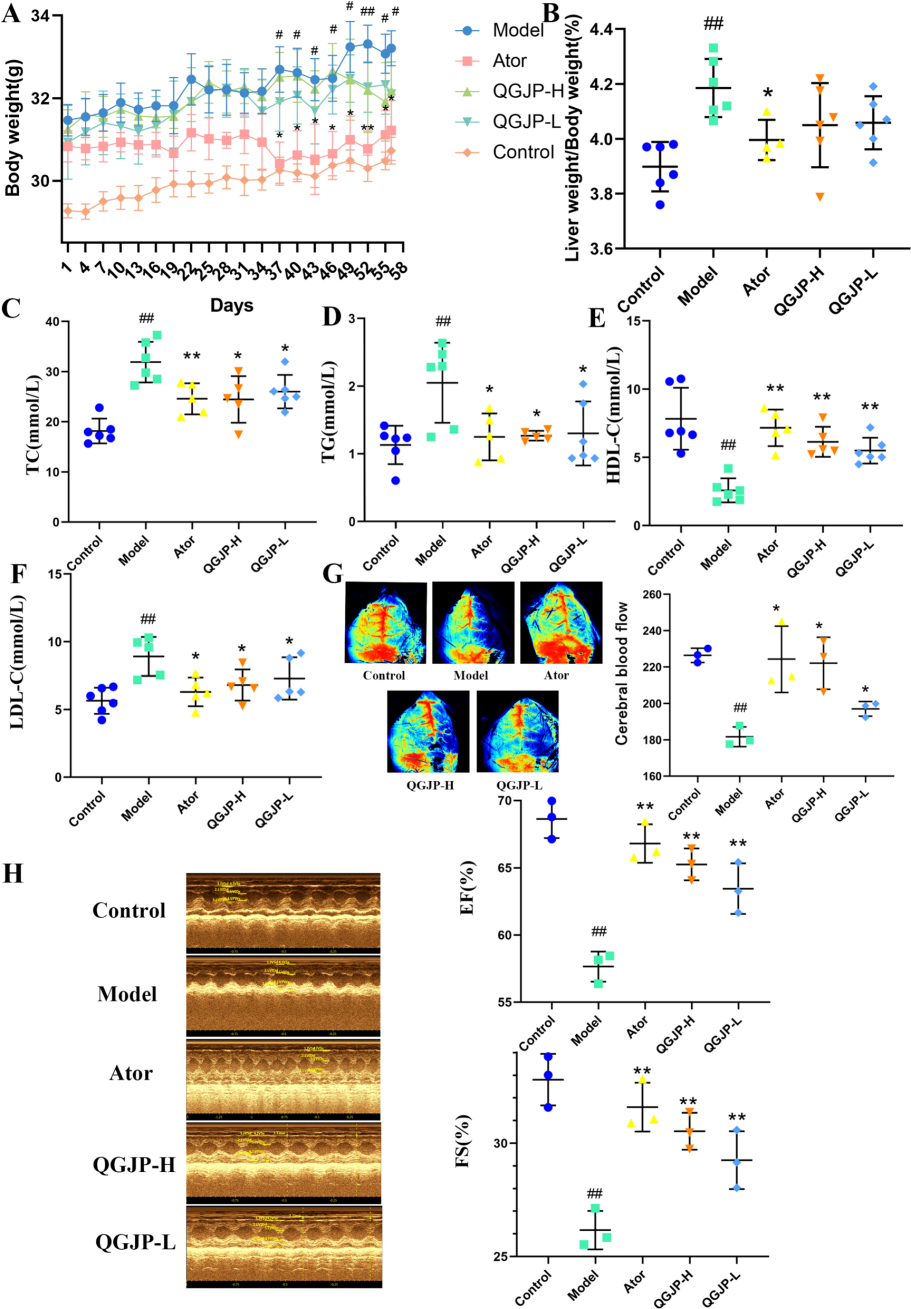
QGJP ameliorates HFD-induced AS and lipid accumulation in mice. (**a**) Body weight of AS mice (x̄ ± s, n = 6). **(b)** Alterations in liver index during AS progression in mice. Liver index = liver weight/body weight (x̄ ± s, n = 6). (**c–f**) Serum TG, TC, LDL and HDL (x̄ ± s, n = 6). (**g**) Changes in cerebral blood flow in mice. (x̄ ± s, n = 3). (**h**) Changes in ejection fraction and fractional shortening in mice. (x̄ ± s, n = 3). ^##^*p* < 0.01 compared with Control group; ^#^*p* < 0.05 compared with Control group; ^**^p < 0.01 compared with Model group; ^*^p < 0.05 compared with Model group

QGJP ameliorates HFD-induced AS and lipid accumulation in mice. AS was induced in ApoE⁻/⁻ mice by 12 weeks of HFD feeding, followed by 8 weeks of QGJP treatment. Throughout the experiment, body weight increased progressively during AS development. At the end of the treatment period, HFD-fed mice exhibited significantly increased body weight and liver index, and HFD-fed ApoE⁻/⁻ mice showed markedly elevated serum levels of total cholesterol (TC), triglycerides (TG), and low-density lipoprotein cholesterol (LDL-C), while high-density lipoprotein cholesterol (HDL-C) was significantly decreased. QGJP treatment reversed these HFD-induced alterations in lipid profiles and improved hyperlipidemia in AS mice (Fig. 1a–f).

Meanwhile, high-fat feeding led to a decline in cardiac function in mice, as shown by decreased ejection fraction and fractional shortening. QGJP improved cardiac function, elevating ejection fraction and fractional shortening and increased total cerebral blood flow (Fig. 1g–h). These results indicate that QGJP effectively alleviates AS symptoms in mice.

### QGJP ameliorates AS pathological injury

**Fig. 2:**
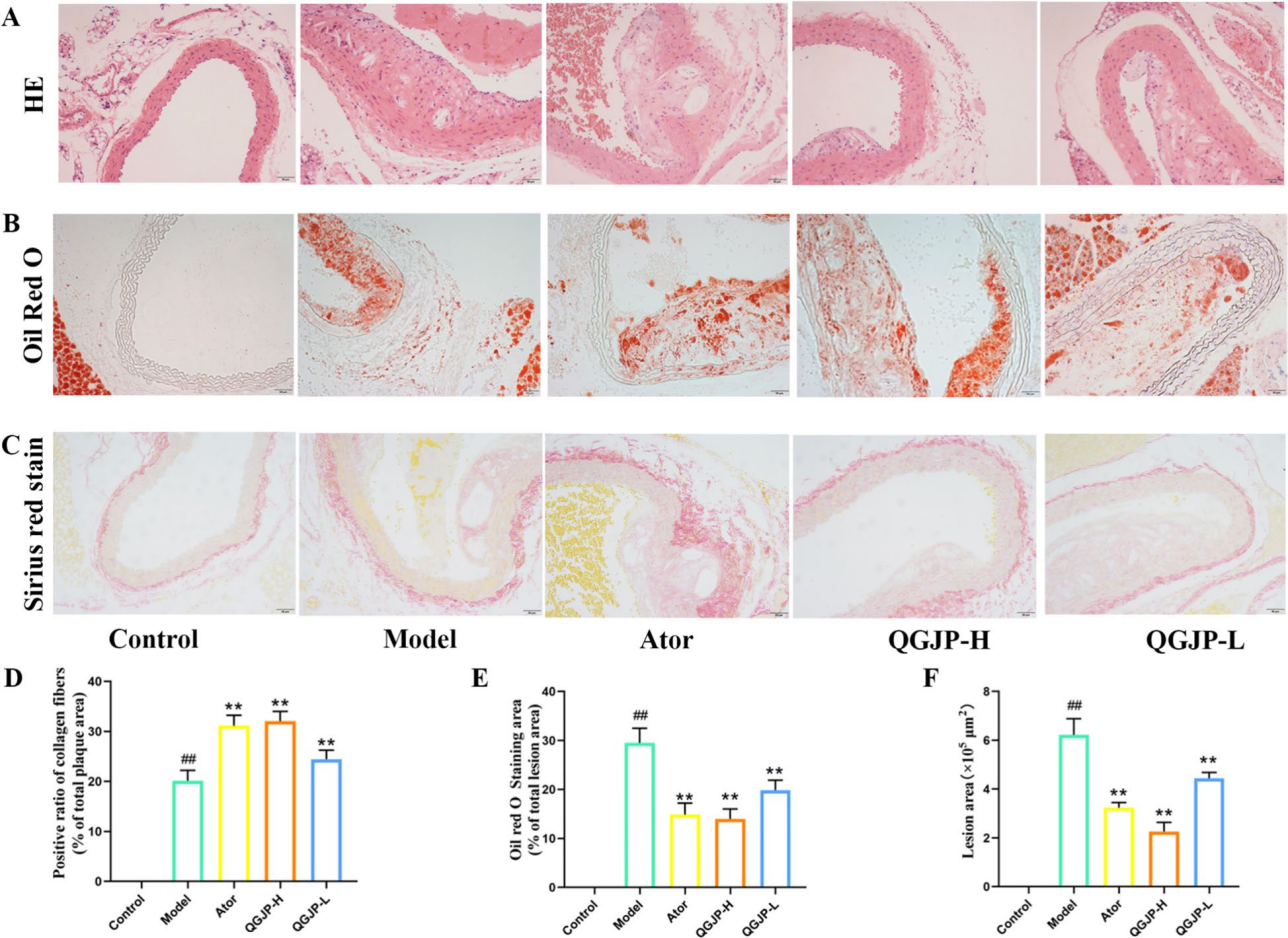
Effects of QGJP on aortic pathological injuries in mice. (**a**) H&E staining was performed to evaluate the effect of QGJP on aortic histopathology in AS mouse. Scale bar: 50 µm (x̄ ± s, n = 3). (**b)** Oil Red O staining was performed to assess the lipid content in the aortic plaques. Scale bar: 50 µm (x̄ ± s, n = 3). (**c**) Sirius Red staining was performed to assess the extent of aortic fibrosis. Scale bar: 50 µm (x̄ ± s, n = 3). (**d**) Positive ratio of collagen fibers measured by Sirius Red staining. (**e**) Oil red O staining area measured by Oil Red O staining. (**f**) Lesion area measured b*p*y H&E staining. . ^##^*p* < 0.01 compared with Control group; ^#^ < 0.05 compared with Control group; ^**^p < 0.01 compared with Model group; ^*^p < 0.05 compared with Model group

QGJP effectively alleviated pathological injury in AS mice. Compared with the normal group, significant intimal thickening was observed in the aorta of mice in the model group, and a large number of AS plaques in the lumen were deposited in the inner wall of the vessel; compared with the model group, the aortic intima of mice in the QGJP low and high dose groups became thinner, the AS plaque area was significantly reduced, and the pathological conditions were significantly improved (Fig. 2a, f). Oil Red O staining revealed minimal lipid deposition in the aortas of the control group, while AS mice displayed substantial lipid accumulation within plaques (Fig. 2b, e). QGJP treatment significantly reduced lipid content in the aorta. Furthermore, Sirius Red staining demonstrated that QGJP increased collagen content and improved its quality and distribution, indicating a stabilizing effect on AS plaques (Fig. 2c, d).

### QGJP alleviates AS-associated endothelial dysfunction

QGJP effectively ameliorated HFD-induced endothelial dysfunction in mice and ox-LDL-induced injury in HUVECs. Given that AS is a chronic inflammatory disease initiated by endothelial disruption, the adhesion of endothelial cells to macrophages represents a pivotal link between initial injury and plaque formation.

**Fig. 3:**
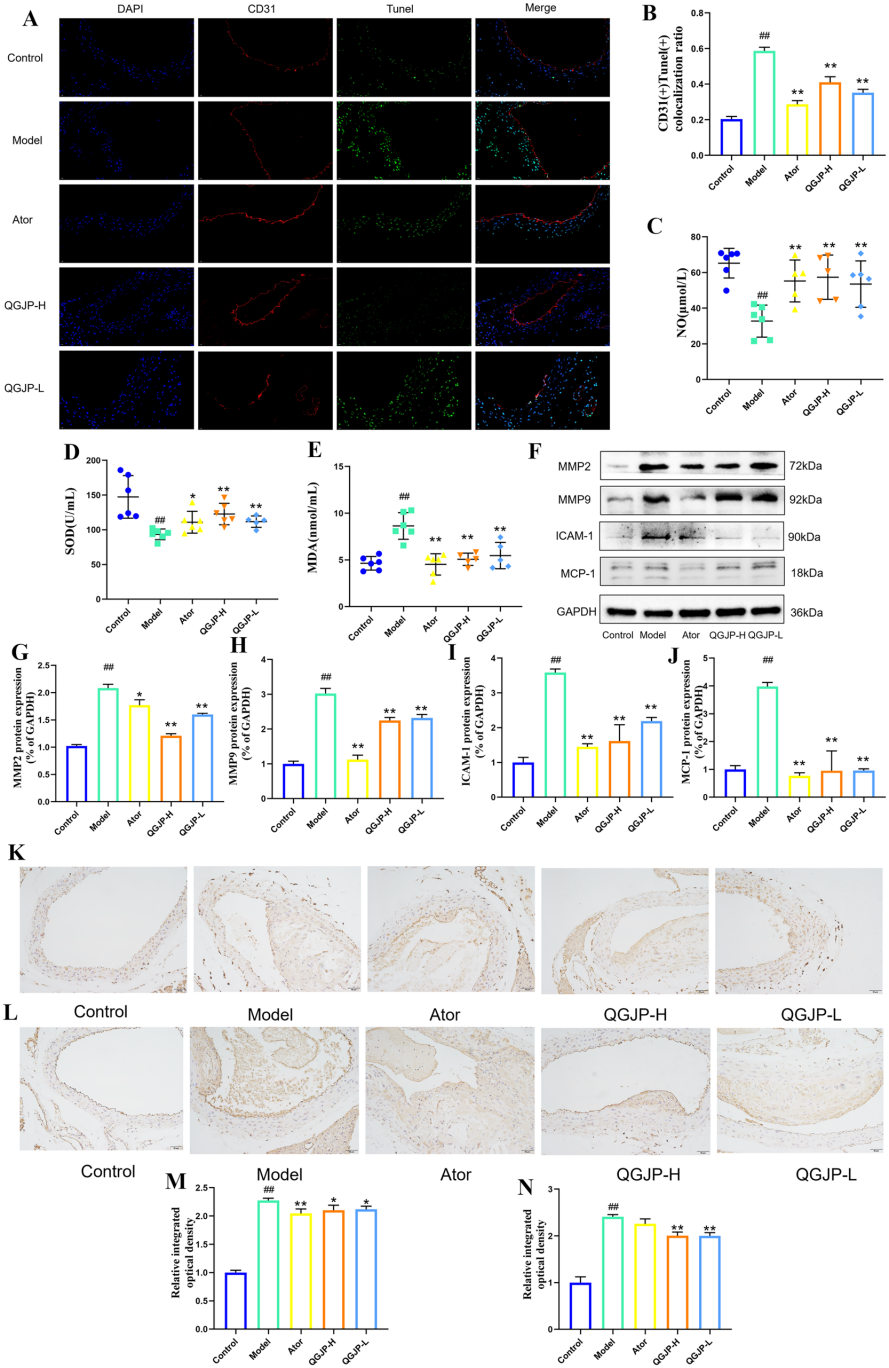
Effects of QGJP on Aortic Endothelial Cell Injury in AS mice. (**a**, **b**) Representative IF images showing the effect of QGJP on endothelial cell apoptosis. Scale bar: 50 µm (x̄ ± s, n = 3). (**c–e**) Serum levels of NO, SOD, and MDA in mice (x̄ ± s, n = 6). (**f–j**) Protein expression levels of MMP2, MMP9, ICAM-1, and MCP-1 in mice aortic tissues, as determined by WB analysis (x̄ ± s, n = 3). (**k–n**) Representative IHC staining showing the expression of MMP2 and ICAM-1 in mice aortic tissues. Scale bar: 50 µm (x̄ ± s, n = 3). ^##^*p* < 0.01 compared with Control group; ^#^*p* < 0.05 compared with Control group; ^**^p < 0.01 compared with Model group; ^*^p < 0.05 compared with Model group

QGJP treatment significantly reduced aortic endothelial cell apoptosis in AS mice, as detected by CD31/TUNEL staining. This was accompanied by a marked amelioration of systemic oxidative stress, evidenced by decreased MDA and increased SOD and NO levels. (Fig. 3a–e). These findings point to the antioxidant and endothelial-protective effects of QGJP.

**Fig. 4:**
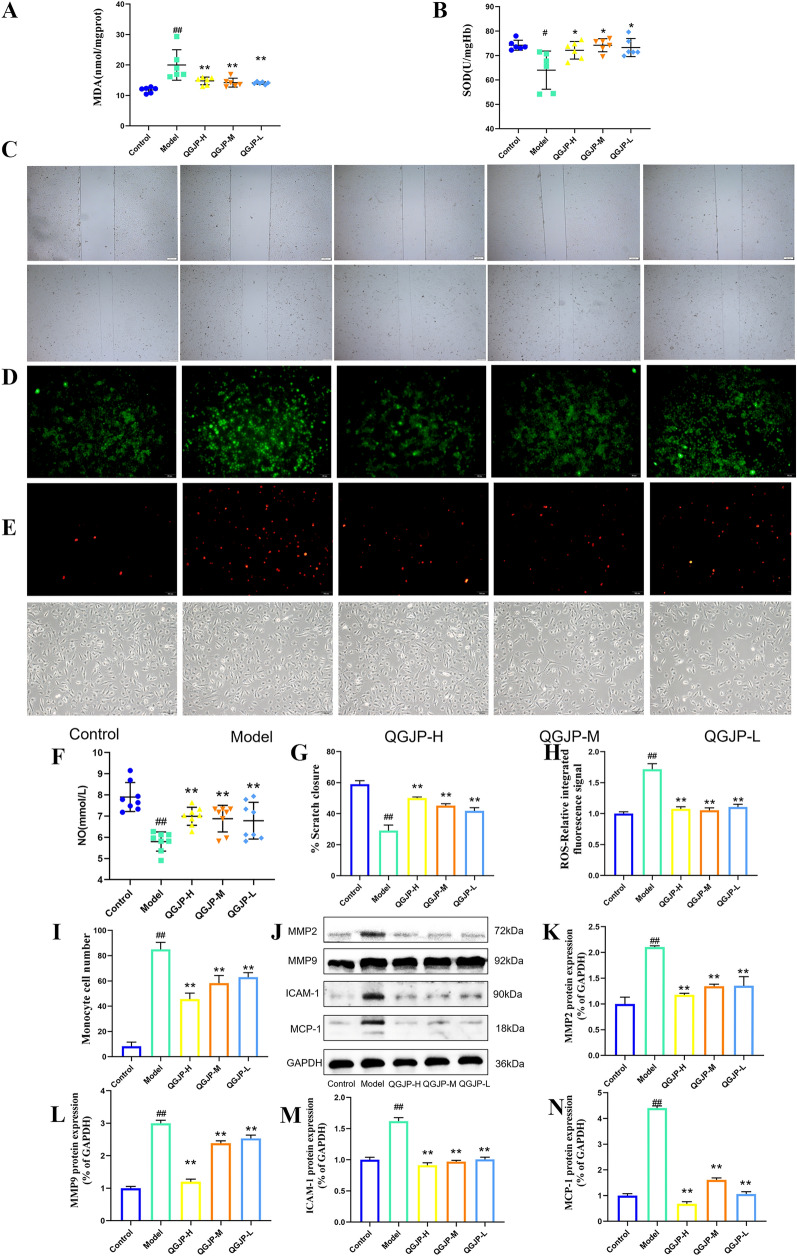
Effects of QGJP on ox-LDL-induced injury in HUVECs. (**a**, **b**, **f**) Intracellular levels of NO, SOD, and MDA in HUVECs (x̄ ± s, n = 6). (**c**, **g**) Representative images from a scratch wound assay showing the migration ability of HUVECs. Scale bar: 200 µm (x̄ ± s, n = 3). (**d**, **h**) Representative immunofluorescence (IF) images detecting intracellular ROS levels. Scale bar: 50 µm (x̄ ± s, n = 3). (**e**, **i**) The number of macrophages adhering to endothelial cells. Scale bar: 50 µm (x̄ ± s, n = 3). (**j-n**) Protein expression levels of MMP2, MMP9, ICAM-1, and MCP-1 in HUVECs (x̄ ± s, n = 3). ^##^*p* < 0.01 compared with Control group; ^#^*p* < 0.05 compared with Control group; ^**^p < 0.01 compared with Model group; ^*^p < 0.05 compared with Model group

Oxidized low-density lipoprotein (ox-LDL) plays a critical role in the pathogenesis of AS. To model this process in vitro, we used ox-LDL to induce endothelial injury in HUVECs. Stimulation with ox-LDL resulted in increased levels of MDA and ROS, alongside a decrease in SOD and NO activity. Treatment with QGJP markedly reversed these alterations, effectively attenuating the ox-LDL-induced lipid injury in HUVECs (Fig. S1, Fig. 4a–h).

A scratch assay demonstrated that QGJP significantly promoted HUVEC migration, which was impaired by ox-LDL stimulation (Fig. 4c, g). Furthermore, QGJP consistently downregulated the key matrix-degrading enzymes MMP-2 and MMP-9, as evidenced by WB in both mouse aortic tissues (Fig. 3f-h) and ox-LDL-treated HUVECs (Fig. 4j-l). These results imply that the inhibition of MMP-driven ECM degradation is a potential mechanism through which QGJP maintains endothelial integrity.

### QGJP inhibition of endothelial adhesion in AS

QGJP significantly attenuated endothelial adhesive capacity, effectively inhibiting the adhesion of macrophages to endothelial cells. Underlying this effect, in a state of chronic, low-grade inflammation, endothelial cells become persistently activated and secrete chemokines that prime monocytes, shifting macrophage surface integrins into a high-affinity state. These high-affinity integrins then mediate firm and irreversible binding to their induced adhesion molecules on the endothelial surface.

To evaluate the impact of QGJP on endothelial adhesion, we first examined the adhesion of THP-1 monocytes to ox-LDL-stimulated HUVECs. The results showed that ox-LDL induction promoted monocyte adhesion, which was significantly suppressed by QGJP treatment (Fig. 4e, i). Further analysis revealed that ox-LDL markedly upregulated the expression of ICAM-1 and MCP-1 in HUVECs, and this induction was effectively counteracted by QGJP (Fig. 4j–n). Consistent with these in vitro findings, QGJP administration also significantly reduced the aortic protein levels of ICAM-1 and MCP-1 in HFD fed ApoE^⁻/⁻^ mice (Fig. 3f–j).

Collectively, these results indicate that lipids induce endothelial injury in AS, primarily by impairing endothelial repair and promoting monocyte adhesion. QGJP conferred protection against this lipid-induced damage, thereby contributing to the attenuation of AS.

### QGJP suppresses macrophage pro-inflammatory polarization

Based on the observed endothelial-monocyte adhesion, we hypothesized that AS progression is closely associated with macrophage activity. Transcriptomic analysis revealed that the disease was significantly enriched in biological processes including inflammatory response, immune response, cell adhesion, and leukocyte cell–cell adhesion, thus validating the significance of targeting macrophages for AS treatment (Fig. S4).

Upon endothelial injury, circulating monocytes are recruited to the vascular wall, where they transmigrate across the endothelial layer and differentiate into macrophages within the subendothelial space. The primary task of these macrophages is to clear deposited lipids, particularly oxidized low-density lipoprotein (ox-LDL). Furthermore, macrophages exhibit high plasticity and can polarize into distinct functional phenotypes in response to different microenvironmental signals, thereby playing diverse and often opposing roles in the disease process.

**Fig. 5:**
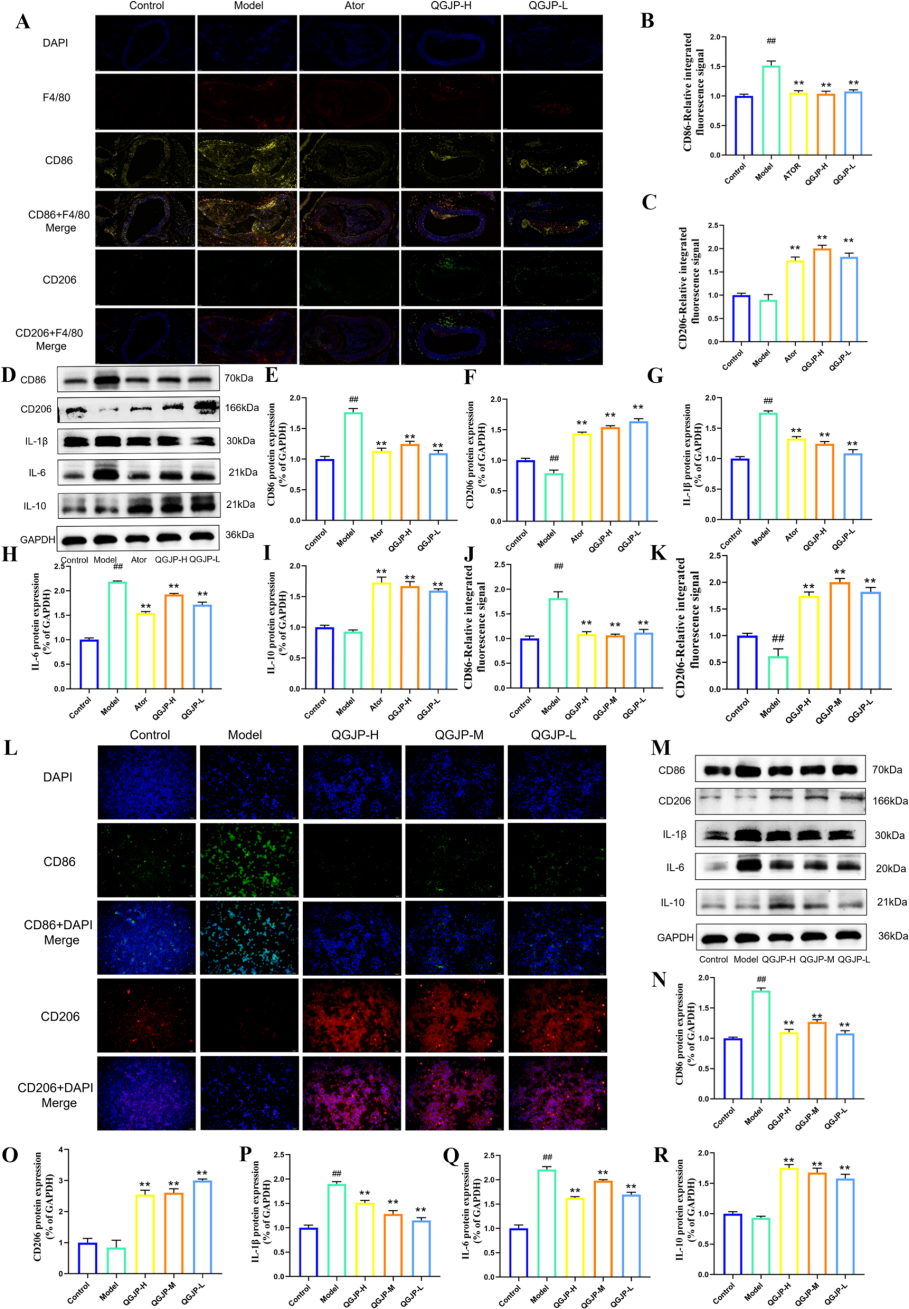
Effects of QGJP on Macrophage Polarization. (**a–c**) Representative immunofluorescence images and quantification of CD86 and CD206 expression in mice aortic tissues (x̄ ± s, n = 3). (**d–k**) Protein expression levels of CD86, CD206, IL-1β, IL-6, and IL-10 in mice aortas, as determined by WB analysis (x̄ ± s, n = 3). (**j–l**) Representative immunofluorescence images and quantification of CD86 and CD206 expression in THP-1 cells (x̄ ± s, n = 3). (**m-r**) Protein expression levels of CD86, CD206, IL-1β, IL-6, and IL-10 in THP-1 cells, as determined by WB analysis (x̄ ± s, n = 3). ^##^*p* < 0.01 compared with Control group; ^#^*p* < 0.05 compared with Control group; ^**^p < 0.01 compared with Model group; ^*^p < 0.05 compared with Model group

In AS mice, QGJP treatment decreased the expression of CD86, IL-1β, and IL-6, while significantly increasing the levels of CD206 and IL-10 (Fig. 5a–k). To model plaque macrophages in vitro, human THP-1 monocytes were stimulated with ox-LDL. Consistent with the in vivo findings, ox-LDL significantly promoted the expression of the M1 marker CD86 and the pro-inflammatory cytokines IL-1β and IL-6, but suppressed the M2 marker CD206 and the anti-inflammatory cytokine IL-10. QGJP treatment effectively reversed these polarizing effects induced by ox-LDL (Fig. 5l–r).

### QGJP treatment induces metabolic in macrophages

The polarization of macrophage phenotypes is closely linked to their metabolic state, with metabolic reprogramming serving as both the foundation and driver of their functional differentiation. Specifically, inflammatory macrophages are highly dependent on glycolysis for energy production, whereas reparative macrophages rely more on oxidative phosphorylation.

**Fig. 6:**
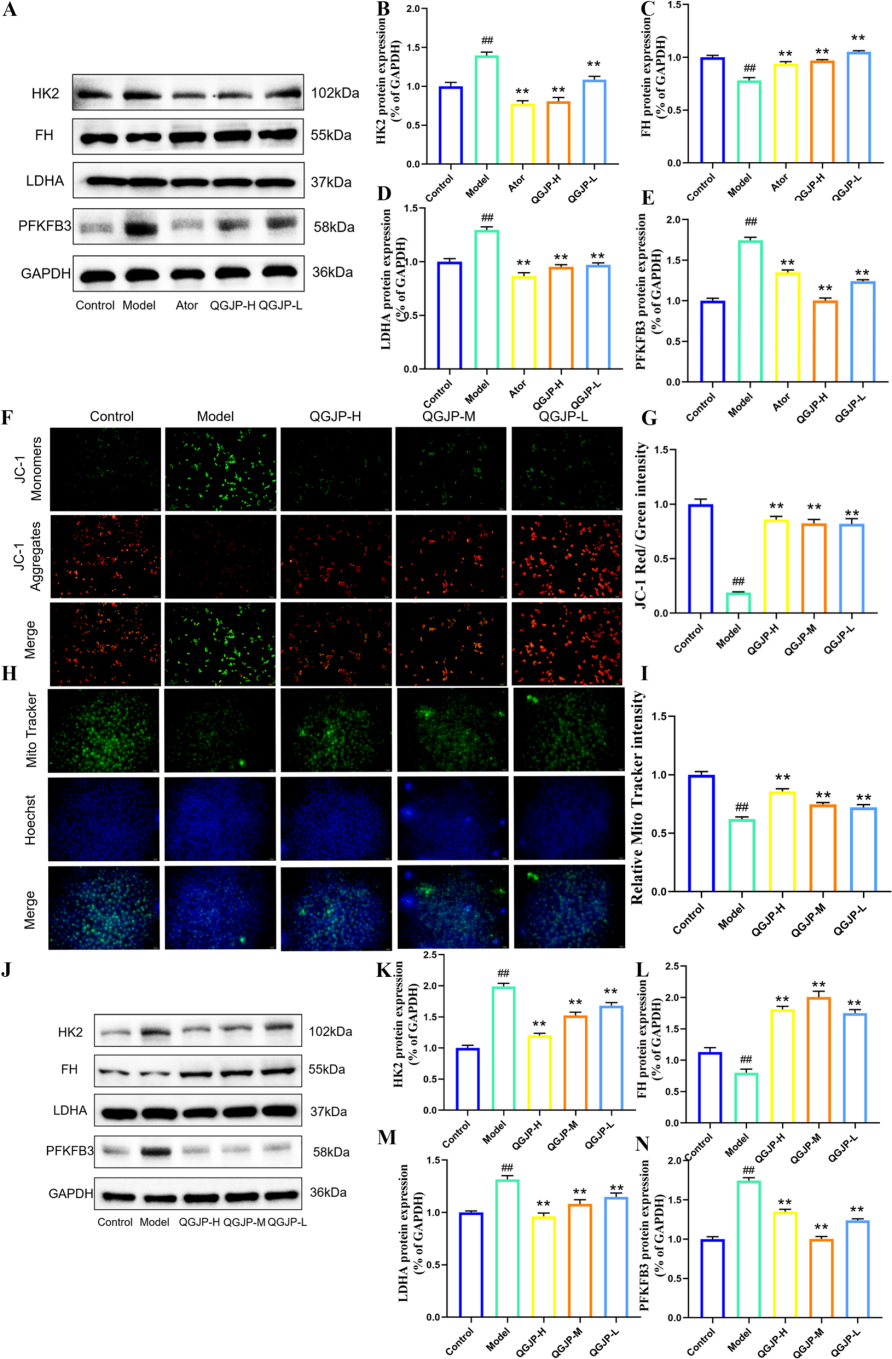
QGJP induces metabolic reprogramming in macrophages. (**a–e**) Protein expression levels of HK2, FH, LDHA, and PFKFB3 in aortic tissues of AS mice, as determined by WB analysis (x̄ ± s, n = 3). (**f–i**) Mitochondrial membrane potential (MMP) was measured in THP-1 cells using JC-1 staining and Mito Tracker staining. A decrease in red fluorescence accompanied by an increase in green fluorescence indicates mitochondrial membrane depolarization. Scale bar: 50 µm (x̄ ± s, n = 3). (**j–n**) Protein expression levels of HK2, FH, LDHA, and PFKFB3 in THP-1 cells, as determined by WB analysis (x̄ ± s, n = 3). ^##^*p* < 0.01 compared with Control group; ^#^*p* < 0.05 compared with Control group; ^**^p < 0.01 compared with Model group; ^*^p < 0.05 compared with Model group

WB analysis revealed that both the AS mice and the ox-LDL-induced macrophage model exhibited decreased expression of FH and increased levels of glycolytic proteins PFKFB3, HK2, LDHA. QGJP treatment suppressed this enhanced glycolysis and promoted oxidative phosphorylation (Fig. 6a–e, j–n). These findings suggest that QGJP modulates macrophage metabolism by reducing glycolysis and enhancing oxidative phosphorylation, which may underlie its inhibition of M1 polarization. Mitochondrial dysfunction, which impairs cellular energy conversion, plays a critical role in AS. Notably, QGJP restored the ox-LDL-induced reduction in mitochondrial membrane potential and loss of mitochondrial mass (Fig. 6f–i).

These results demonstrate that QGJP suppresses inflammatory (M1) polarization and promotes reparative (M2) polarization in macrophages by modulating their cellular energy metabolism.

### QGJP modulation of macrophage histone lactylation

**Fig. 7:**
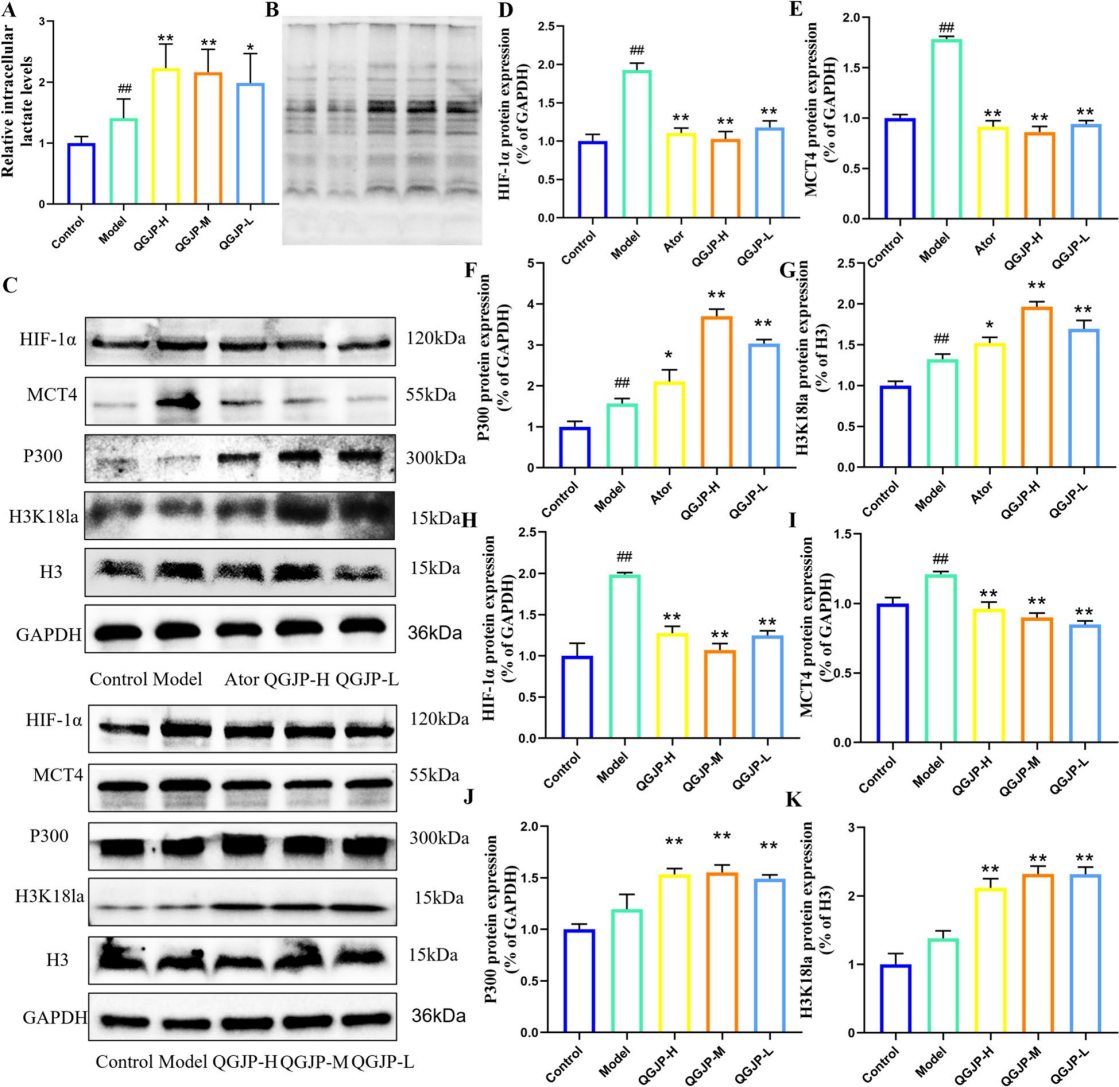
QGJP regulates histone lactylation in macrophages. (**a**) Intracellular lactate concentration in ox-LDL-induced THP-1 cells (x̄ ± s, n = 6). (**b**) Immunoblots for Pan Kla in aortic tissues (x̄ ± s, n = 3). (**c–g**) Protein expression levels of HIF-1α, MCT4, P300, and H3K18la in aortic tissues of AS mouse, as determined by WB analysis (x̄ ± s, n = 3). (**h**–**k**) Protein expression levels of HIF-1α, MCT4, P300, and H3K18la in THP-1 cells, as determined by WB analysis (x̄ ± s, n = 3). ^##^*p* < 0.01 compared with Control group; ^#^*p* < 0.05 compared with Control group; ^**^p < 0.01 compared with Model group; ^*^p < 0.05 compared with Model group

**Fig. 8:**
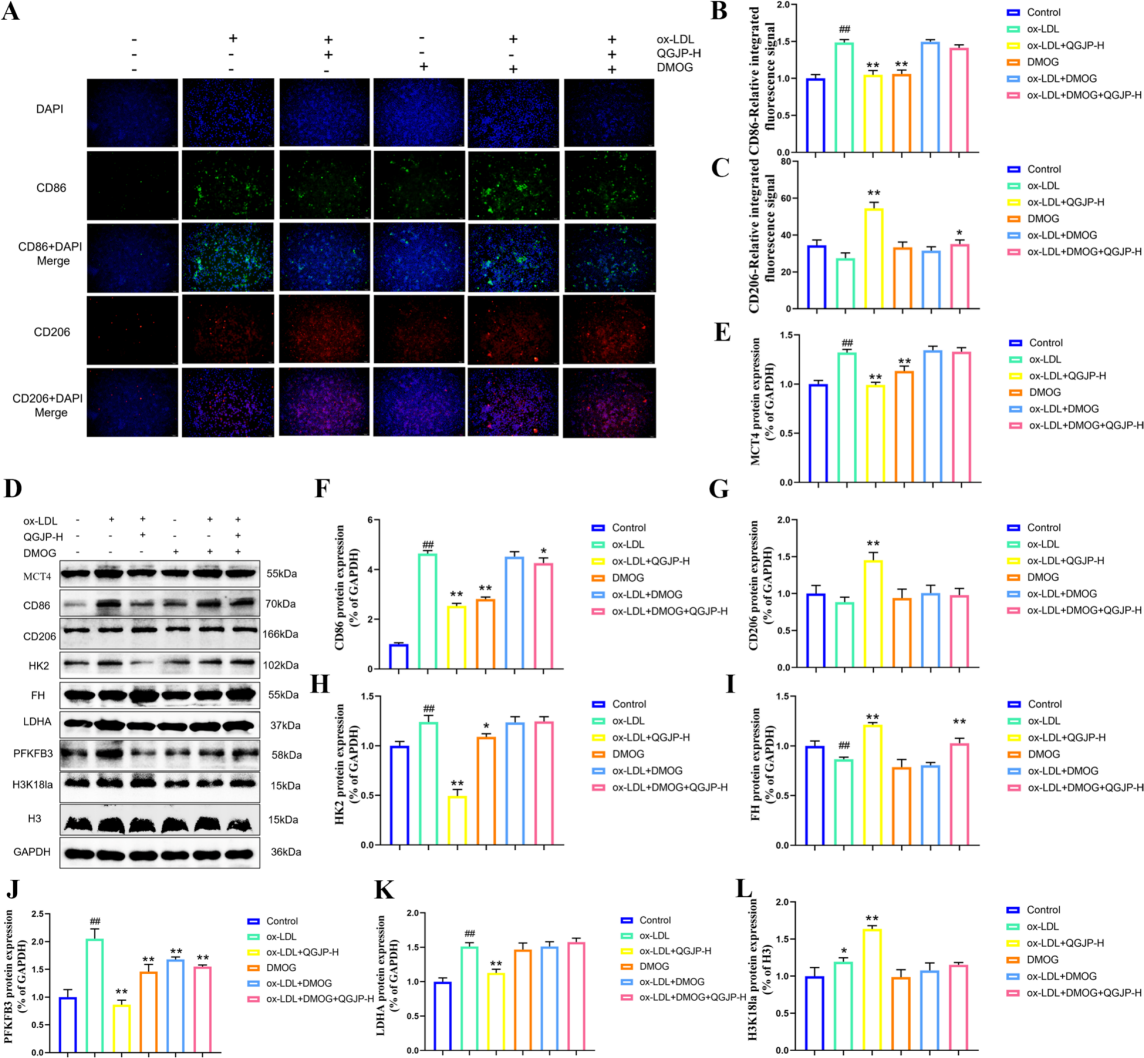
DMOG treatment attenuates the regulatory effects of QGJP on macrophage polarization. (**a–c**) Representative immunofluorescence (IF) images showing the effect of QGJP on macrophage polarization following DMOG treatment. Scale bar: 50 µm (x̄ ± s, n = 3). (**d–l**) Protein expression levels of macrophage polarization markers following DMOG and QGJP treatment, as determined by WB analysis (x̄ ± s, n = 3). ^##^*p* < 0.01 compared with Control group; ^#^*p* < 0.05 compared with Control group; ^**^p < 0.01 compared with Model group; ^*^p < 0.05 compared with Model group

Upon polarization to a pro-inflammatory phenotype, macrophages undergo a metabolic shift toward glycolysis, resulting in substantial lactate production, even under aerobic conditions. This lactate is not merely a metabolic byproduct but also functions as an important signaling molecule. Our study revealed that both the AS mouse model and the ox-LDL-induced macrophage polarization model exhibited significantly elevated protein levels of the lactate transporter MCT4 and its upstream regulator HIF-1α, a trend that was effectively reversed by QGJP treatment (Fig. 7c–e, h–i) (Fig. S3). To further investigate the role of lactate transport in QGJP-mediated modulation of macrophage polarization, we treated THP-1 macrophages with DMOG, a HIF-1α agonist. DMOG treatment abolished the inhibitory effect of QGJP on MCT4 expression. Correspondingly, DMOG also mitigated the regulatory effects of QGJP on macrophage polarization and energy metabolism (Fig. 8a–k). These results suggest that the mechanism by which QGJP modulates macrophage polarization may involve the HIF-1α/MCT4 axis-regulated lactate transport.

Following MCT4 inhibition, lactate accumulates intracellularly to high concentrations. This elevated lactate is not merely a metabolic waste product but also serves as a crucial signaling precursor. It enters the nucleus and acts as a direct substrate for histone lactylation, leading to the modification of histones by specific enzymes. Histone lactylation is a recently discovered key mechanism that directly links cellular metabolism to epigenetic regulation and immune cell function.

Measurement of intracellular lactate levels in THP-1 macrophages revealed that ox-LDL induction led to a modest increase, whereas QGJP treatment significantly elevated lactate content compared to the model group (Fig. 7a). This accumulation of intracellular lactate may be associated with decreased MCT4 expression. Based on pan-Kla analysis in ox-LDL-induced THP-1 cells, QGJP treatment was found to increase the overall lactylation level. Further detection revealed enhanced lactylation at approximately 15 kDa, suggesting that histone H3 may be a potential target of lactylation modification. WB analysis demonstrated that QGJP treatment markedly increased the expression levels of both H3K18la and p300 (Fig. 7b, f–g, j–k). These results suggest that QGJP may modulate macrophage polarization by promoting histone H3K18 lactylation. Furthermore, when THP-1 macrophages were co-treated with the HIF-1α agonist DMOG, the enhancing effect of QGJP on H3K18la was abolished (Fig. 8l). Collectively, these findings indicate that QGJP likely regulates macrophage phenotypic switching through the HIF-1α/MCT4-mediated lactate transport pathway, thereby facilitating histone H3K18 lactylation to exert its immunomodulatory effects.

### Analysis of bioactive blood components of QGJP and molecular docking

Our previous experiments demonstrated that QGJP exerts anti-AS effects both in vivo and in vitro by suppressing M1 macrophage polarization through the modulation of histone lactylation. However, the precise bioactive constituents responsible for these therapeutic effects remain uncharacterized.

**Fig. 9:**
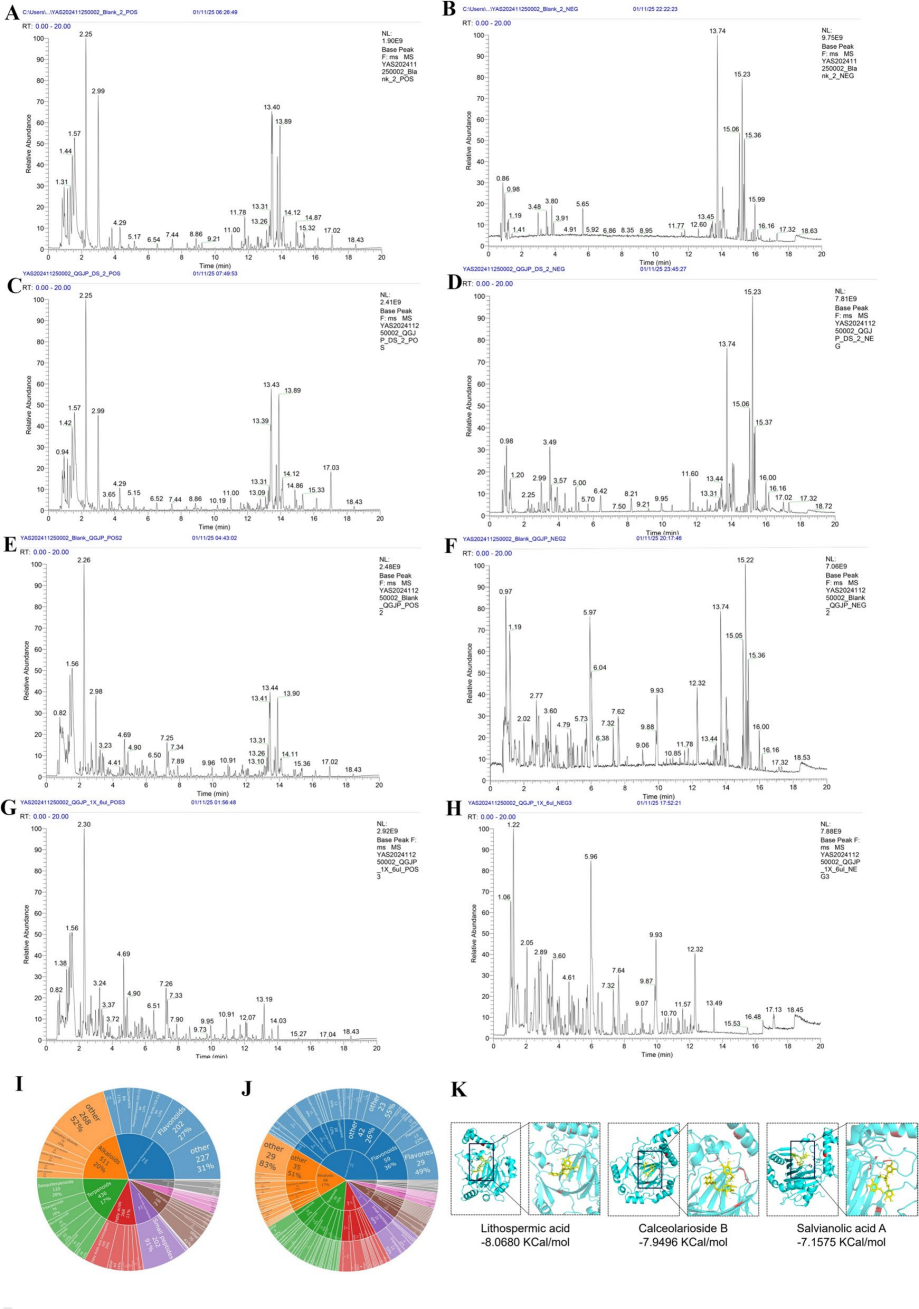
The standard ion chromatogram of QGJP analysis using the UHPLC-HRMS method. (**a–b**) The positive ion mode and the negative ion mode of Control serum in UHPLC-HRMS analysis. (**c–d**) The positive ion mode and the negative ion mode of QGJP-containing serum in UHPLC-HRMS analysand. (**e**–**f**) The positive ion mode and the negative ion mode of QGJP + Control serum in UHPLC-HRMS analysis. (**g–h**) The positive ion mode and the negative ion mode of QGJP in UHPLC-HRMS analysis. (**i–j**) The distribution of QGJP compounds and QGJP-containing serum compounds across chemical classes. (**k**) Molecular docking between compounds SAA, LA, and CAB and HIF-1α

To further elucidate the bioactive basis of QGJP, we performed UHPLC-HRMS analysis to identify its primary active components absorbed into the bloodstream. A total of 47 compounds derived from QGJP were identified in the serum (Fig. 9a–j) (Table. S2). Based on the consistent efficacy observed in prior in vivo and in vitro studies, we employed molecular docking to predict the interactions between these absorbed components and HIF-1α. By evaluating binding affinity and the number of hydrogen bonds, compounds SAA, LA, and CAB were predicted as the key constituents potentially responsible for mediating the effects of QGJP on macrophage polarization (Fig. 9k).

### Effects of SAA, LA, and CAB on M1 macrophage polarization and lactylation

**Fig. 10:**
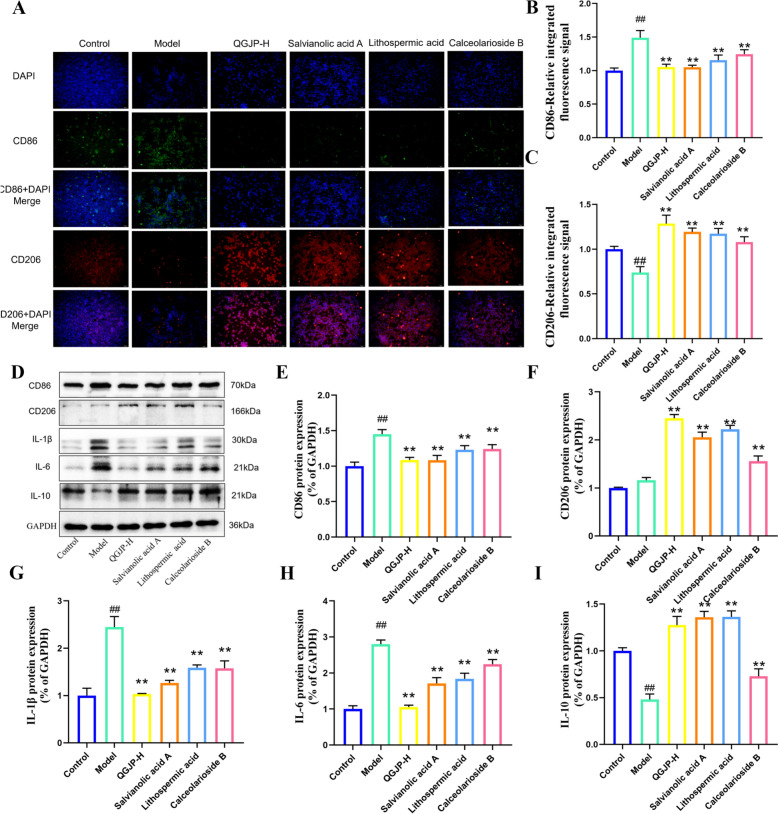
Effects of SAA, LA, and CAB on M1 Macrophage Polarization. (**a–c**) Representative immunofluorescence (IF) images showing the effects of SAA, LA, and CAB on macrophage polarization. Scale bar: 50 µm (x̄ ± s, n = 3). (**d–i**) Protein expression levels of macrophage polarization markers following treatment with SAA, LA, and CAB, as determined by WB analysis (x̄ ± s, n = 3). ^##^*p* < 0.01 compared with Control group; ^#^*p* < 0.05 compared with Control group; ^**^p < 0.01 compared with Model group; ^*^p < 0.05 compared with Model group

Next, we treated ox-LDL-induced macrophages with SAA (100 μM), LA (50 μM), and CAB (25 μM) (Fig. S2). The results showed that all three compounds significantly suppressed the protein expression of CD86, IL‑1β, and IL‑6, while promoting the expression of CD206 and IL‑10 (Fig. 10a–i). These findings confirm that SAA, LA, and CAB effectively inhibit the polarization of macrophages toward a pro‑inflammatory phenotype.

**Fig. 11:**
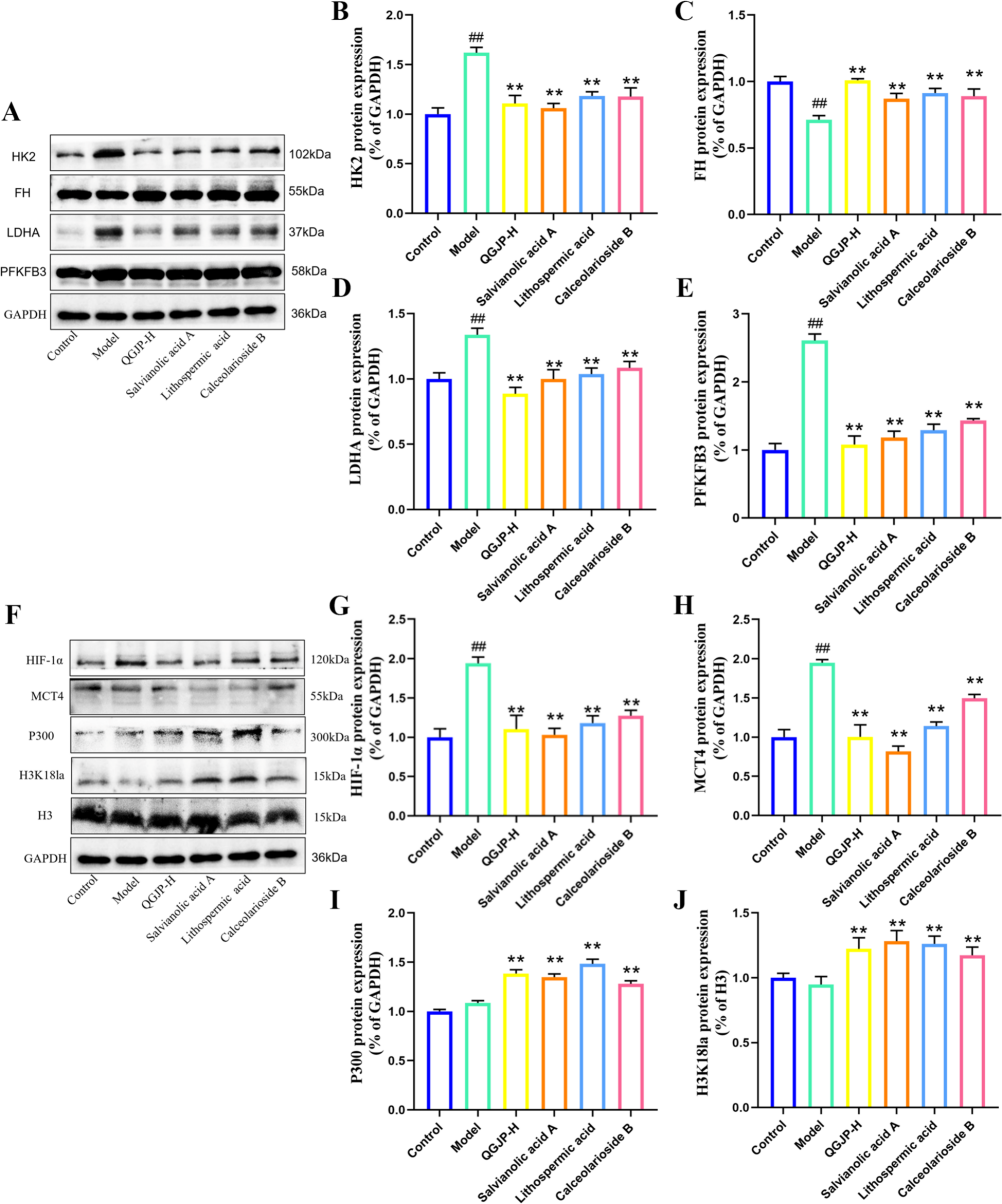
Effects of SAA, LA, and CAB on macrophage glycolysis and histone lactylation.(**a–e**) Protein expression levels of key glycolytic and oxidative phosphorylation markers in macrophages treated with SAA, LA, and CAB, as determined by WB analysis (x̄ ± s, n = 3). (**f–j**) Protein expression levels of histone lactylation-related proteins in macrophages treated with SAA, LA, and CAB, as determined by WB analysis (x̄ ± s, n = 3). ^##^*p* < 0.01 compared with Control group; ^#^*p* < 0.05 compared with Control group; ^**^p < 0.01 compared with Model group; ^*^p < 0.05 compared with Model group

Subsequent analysis of proteins related to glycolysis and oxidative phosphorylation revealed that SAA, LA, and CAB significantly suppressed the expression of CD86, IL-1β, LDHA, PFKFB3, and HK2, while promoting the expression of CD206, IL-10, FH, MCT4, p300, and H3K18la (Fig. 11a–j). These results suggest that SAA, LA, and CAB are likely the key active constituents through which QGJP inhibits M1 macrophage polarization and alleviates AS.

## Discussion

In recent years, research on the pathogenesis and treatment strategies of AS has shifted from traditional risk factors to emerging areas such as immunometabolic regulation and epigenetic modifications [1]. Considering the well-established safety of TCM in long-term clinical use, they are increasingly recognized for their potential in treating AS. In this context, the present study aims to clarify the pharmacological effects of QGJP and its active components against AS, with a particular focus on their role in regulating the “metabolism-epigenetics axis” in macrophages. Our goal is to establish a functional link between metabolic reprogramming and epigenetic remodeling in the context of TCM-based interventions for AS.

Using a combined strategy of a HFD ApoE^−/−^ mouse model of AS and an ox-LDL stimulated endothelial cell model, we demonstrated at different levels that QGJP significantly ameliorated AS symptoms. Subsequent transcriptomic analysis and investigation into the pathogenesis of AS suggested a strong association between the anti-AS effects of QGJP and endothelial cell adhesion, inflammation, and immune regulation. Further examination of macrophage phenotype and function-related proteins revealed that QGJP suppressed M1 macrophage polarization while promoting the expression of repair-related genes. Additionally, QGJP inhibited the expression of key glycolytic proteins and enhanced FH expression. In-depth mechanistic investigation demonstrated that QGJP, by inhibiting the HIF-1α/MCT4 axis and its mediated lactate efflux, promoted histone lactylation. This epigenetic modification activated the transcription of anti-inflammatory genes and TCA cycle-related genes, promoting tissue repair and metabolic homeostasis. Finally, we identified 47 absorbed bioactive components in QGJP using UHPLC-HRMS, primarily derived from *Salvia miltiorrhiza* Bunge, *Astragalus membranaceus* (Fisch.) Bunge, *Plantago asiatica* L, and *Crataegus pinnatifida* Bunge—herbs known for their anti-AS and anti-inflammatory properties. Through further molecular docking and experimental validation, we propose that SAA, LA, and CAB are likely the key active constituents responsible for QGJP’s therapeutic effects against AS, mediated through the promotion of histone lactylation and subsequent regulation of macrophage polarization.

The progression of AS involves multiple key processes, including endothelial injury and dysfunction, lipid deposition and oxidative modification, foam cell formation and fatty streak development, smooth muscle cell migration and plaque progression, as well as plaque instability and rupture [19]. Endothelial injury and dysfunction represent the initial stage of AS. Under hyperlipidemic and hyperglycemic conditions, endothelial permeability increases, accompanied by enhanced adhesiveness and upregulated expression of adhesion molecules, which promote the attachment of circulating monocytes and lymphocytes to the vascular wall. Concurrently, nitric oxide (NO) secretion decreases, leading to impaired vasodilation, while chemokines such as MCP-1 are secreted, guiding leukocyte transmigration across the endothelium [20]. Studies have shown that ameliorating endothelial dysfunction can effectively reduce macrophage recruitment and alleviate AS [21, 22]. In our study, the HFD fed ApoE^⁻/⁻^ mouse model exhibited significant vascular endothelial injury, characterized by impaired migration capacity, upregulation of adhesion molecules, enhanced monocyte adhesion, and increased secretion of MCP-1. These findings indicate that enhanced endothelial-monocyte interaction serves as an early pathological basis for AS. Consistent results were observed in ox-LDL-induced HUVECs. Notably, QGJP treatment markedly alleviated endothelial injury. These results suggest that QGJP mitigates endothelial injury and dysfunction by inhibiting endothelial apoptosis and suppressing the expression of adhesion molecules and chemokines.

Notably, endothelial dysfunction promotes extensive monocyte adhesion and facilitates their activation and differentiation into macrophages [23]. Macrophages are among the most abundant immune cells in AS plaques and exhibit remarkable functional plasticity. They can polarize into distinct phenotypes in response to local microenvironmental signals such as cytokines, chemokines, and lipids. Accumulating evidence supports macrophage polarization as a critical target for alleviating AS [21]. In our in vivo and in vitro models of AS, we observed significantly elevated levels of CD86 and pro-inflammatory cytokines, indicating a predominance of M1 macrophages. Following QGJP treatment, the expression of CD86, IL-1β, and IL-6 was suppressed, while the levels of CD206 and IL-10 were markedly increased. These results demonstrate that QGJP inhibits M1 polarization and promotes M2 polarization in AS plaques, thereby restoring the balance between pro-inflammatory and reparative macrophages. Our findings that QGJP modulates macrophage polarization suggest that targeting macrophage function with TCM holds substantial potential for the treatment of AS.

The distinct immune phenotypes of macrophages are closely linked to their intrinsic metabolic processes, with specific metabolic pathways driving and sustaining their functional states [24]. Metabolism serves as the foundation of cellular function, while function reflects its metabolic state. Targeting the metabolic network of macrophages has emerged as a key strategy for reshaping their immunophenotype [25]. Metabolic reprogramming in macrophages refers to the fundamental and coordinated shifts in intracellular metabolic pathways upon activation by external stimuli. These changes supply the necessary energy, biosynthetic precursors, and signaling molecules required for activation, thereby determining and maintaining their specific immune functions [25]. Pharmacological modulation of specific metabolic pathways offers a promising strategy for precisely reprogramming macrophage polarization, thereby steering the course of immune responses. Supporting this notion, studies have demonstrated that treatment with the glycolytic inhibitor 2-DG significantly suppresses the production of pro-inflammatory cytokines, and NO synthesis in LPS-induced M1 macrophages. Moreover, it may indirectly promote the expression of M2-associated genes, possibly through induced energy stress [26]. In chronic inflammatory diseases such as AS, metabolic reprogramming in macrophages is not merely a shift in energy supply, but a central mechanism regulating their functional phenotype [27, 28]. Modulating macrophage metabolism to suppress the M1 phenotype has been recognized as a promising therapeutic strategy against AS. In this study, we observed that under AS conditions, macrophages rapidly upregulate glycolysis and convert pyruvate to lactate via LDHA, leading to significant intracellular lactate accumulation. Treatment with QGJP notably reduced glycolytic activity in macrophages, protected mitochondrial function, enhanced oxidative phosphorylation, and suppressed M1 polarization.

Lactate, as a key glycolytic metabolite, is closely associated with macrophage phenotypic switching and metabolic activity. It influences macrophage polarization through multiple mechanisms, ultimately suppressing inflammatory M1 activation while promoting reparative M2 polarization [29]. In tumor microenvironments, lactate drives M2-like polarization in macrophages, promoting angiogenesis and significantly contributing to immune escape—a hallmark of cancer progression. Furthermore, lactate induces the expression of VEGF and Arg-1, reinforcing the M2-polarized state in macrophages [30, 31]. Within AS plaques, lactate is primarily produced by highly glycolytic M1 macrophages and vascular cells. It contributes to the pathological environment through microenvironment acidification, functional modulation of cells, and histone modification, among other mechanisms. In our experiments, QGJP treatment increased intracellular lactate levels in macrophages, which may be related to altered lactate transport. Under pathophysiological conditions, lactate shuttling between cells and tissues depends on cell-specific lactate transporters. MCT4 is highly expressed in cells and tissues with high glycolytic flux. Inflammatory macrophages significantly upregulate MCT4 expression upon activation to rapidly export lactate generated via glycolysis. Our study demonstrates that QGJP intervention significantly downregulates the expression of HIF-1α in M1-polarized macrophages, consequently suppressing MCT4 protein expression. The reduction in MCT4 disrupts the high-glycolysis metabolic state essential for sustaining the M1 phenotype, thereby effectively inhibiting M1 polarization. Notably, this metabolic alteration also promotes the transition of macrophages toward the reparative M2 phenotype [32]. Furthermore, upregulation of MCT4 expression via DMOG-induced HIF-1α activation antagonized the regulatory effect of QGJP on macrophage polarization, further validating the molecular mechanism by which QGJP modulates macrophage polarization.

Targeting the metabolic products of M1 macrophages to modulate their self-sustaining inflammatory loops and drive phenotypic switching represents a key strategy in regulating macrophage polarization. Studies have revealed that changes in metabolite concentrations resulting from metabolic reprogramming can directly act as substrates or cofactors for posttranslational modifications, enabling precise regulation of reparative/anti-inflammatory gene transcription programs. In contemporary scientific research, the functional role of metabolites has expanded beyond their traditional metabolic activities, demonstrating increasingly broad biological regulatory functions. Notably, metabolites can serve as direct substrates or essential cofactors for epigenetic modifications, such as histone methylation, acetylation, and lactylation-thereby enabling precise control over the epigenetic state and influencing cell fate and function [33, 34]. This finding highlights the intricate network connecting metabolic reprogramming and epigenetic regulation. Our results demonstrate that modulating lactate transport effectively drives macrophage phenotypic polarization, thereby providing a novel therapeutic perspective for intervening in AS and other chronic inflammatory diseases through the “immunometabolic-epigenetic” axis.

In the AS microenvironment, lactate-the primary glycolytic product of macrophages, serves as a substrate for lactylation, a recently identified form of post-translational modification. This process involves the covalent attachment of lactate molecules to lysine residues on histones. The addition of a lactyl group to lysine residues alters protein structure and function [35]. Histone H3K18 lactylation represents a significant epigenetic mechanism. Catalyzed by p300, it is a novel histone modification that has been shown to activate genes associated with wound healing and endotoxin tolerance in macrophages [36, 37]. The Warburg effect in macrophages directly generates lactate as its metabolic endpoint. Enhanced glycolysis inevitably leads to lactate accumulation, which in turn drives histone H3K18 lactylation, thereby functionally linking cellular metabolism to gene expression [38]. These lactylation modifications are specifically enriched in the promoter regions of genes related to M2-like phenotypes and tissue repair [39]. In our study, we found that histone lactylation plays an important role in promoting the transition of macrophages toward a reparative phenotype. Both QGJP and its active constituents significantly enhanced histone lactylation in macrophages, contributing to the alleviation of AS.

Thus, lactylation acts as an endogenous braking mechanism during the later stages of M1 polarization. By promoting the expression of anti-inflammatory and reparative genes, it helps to resolve inflammatory responses in a timely manner and prevents excessive tissue damage. The gene programs upregulated via lactylation drive macrophages toward immunomodulatory, tissue-repairing, and pro-angiogenic functions. In AS, lactylation of M1-like macrophages facilitates their transition to an M2-like phenotype, thereby attenuating inflammatory responses [14]. In this study, we demonstrated that QGJP suppresses lactate efflux, promotes intracellular lactate accumulation, and enhances histone H3K18 lactylation in macrophages—ultimately exerting therapeutic effects against AS. These findings provide new insight into the mechanism by which TCM may ameliorate AS progression. Although this study identifies MCT4 as a key mediator of the anti-atherosclerotic effects of QGJP, the absence of a specific MCT4 inhibitor (e.g., VB124) as a direct comparator limits the specificity of the mechanistic conclusions. MCT4 plays a central role in macrophage lactate transport, and its functional contribution to the observed effects warrants further validation using targeted pharmacological intervention. Future studies incorporating MCT4-specific inhibitors alongside QGJP treatment will be essential to conclusively establish MCT4 as a critical therapeutic target and to strengthen the evidence for lactate transport modulation as a promising strategy against atherosclerosis. Furthermore, although both cellular and animal models were utilized, atherosclerosis is a chronic disease involving a complex microenvironment. The present work primarily focused on macrophages and endothelial cells, and did not systematically examine intercellular crosstalk within a more integrated pathophysiological context in vivo. This may, to some extent, limit the generalizability of the conclusions to the disease as a whole.

## Conclusions

**Fig. 12:**
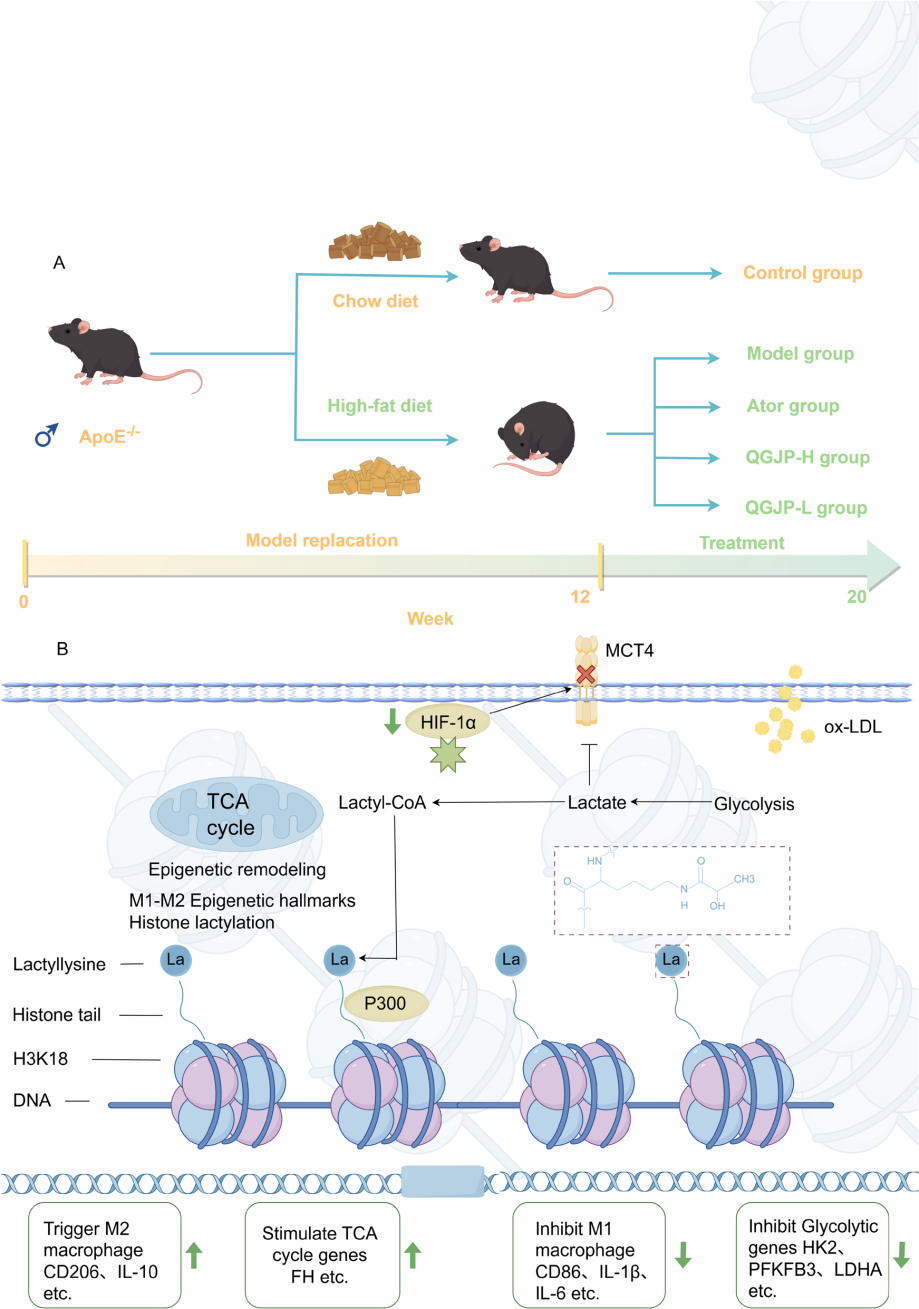
The molecular mechanism of QGJP improving AS

In conclusion, this study establishes the critical role of metabolic reprogramming and epigenetic regulation in AS progression. Our findings suggest that a mechanism whereby QGJP alleviates AS may involve the inhibition of the HIF-1α/MCT4 axis and lactate transport, which regulates histone H3K18 lactylation to promote a reparative macrophage phenotype. (Fig. 12). Collectively, our work highlights the therapeutic potential of targeting the metabolite-epigenetic axis to reprogram immune cell function in cardiovascular disease.

## Supplementary Information

Supplementary Material 1

## Data Availability

The datasets used and/or analyzed during the current study are available from the corresponding author on reasonable request.

## Abbreviations

AS: Atherosclerosis
QGJP: Qinggan Jianpi Formula
HFD: High-fat diet Ox-LDL: oxidized LDL
SAA: Salvianolic acid A
LA: Lithospermic acid
CAB: Calceolarioside B
HIF-1α: Hypoxia-inducible factor 1 alpha
MCT4: Monocarboxylate transporter 4
FH: Fumarate hydratase
PFKFB3: 6-Phosphofructo-2-kinase/fructose-2,6-biphosphatase 3
LDHA: Lactate dehydrogenase A
HK2: Hexokinase 2
CD86: Cluster of differentiation 86
CD206: Macrophage mannose receptor 206
H3K18: Histone H3, lysine 18
ICAM-1: Intercellular adhesion molecule-1
MMP2: Matrix metalloproteinase-2
MMP9: Matrix metalloproteinase-9
MCP-1: Monocyte chemoattractant protein-1
H3: Histone H3
OXPHOS: Oxidative phosphorylation
UHPLC-HRMS: Ultra-high performance liquid chromatography coupled with high-resolution mass spectrometry
WB: Western blot
HUVECs: Human umbilical vein endothelial cells
THP-1: Human acute monocytic leukemia cell line
TCM: Traditional Chinese Medicine
