# Supplementary material for: Qinggan Jianpi formula attenuates atherosclerosis by suppressing macrophage lactate transport to activate repair genes via H3K18 lactylation
Source: Chin Med. 2026 Apr 24;21:121. doi: 10.1186/s13020-026-01394-0 (PMC13107618; doi:10.1186/s13020-026-01394-0)
Supplement: Supplementary file 1 — Supplementary Material 1 [file 13020_2026_1394_MOESM1_ESM.docx]

**S1. The protective effect of QGJP against ox-LDL-induced reduction in HUVECs viability.**

| 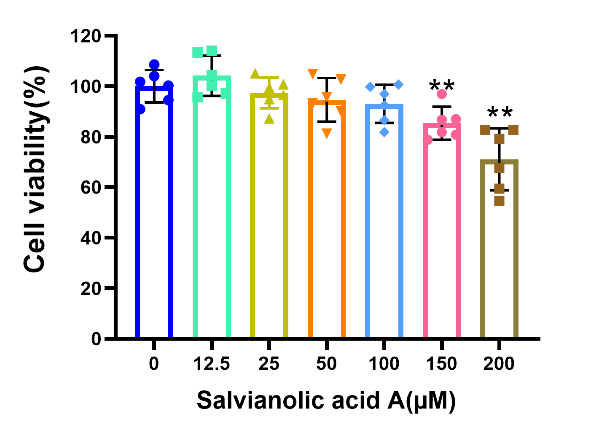 | 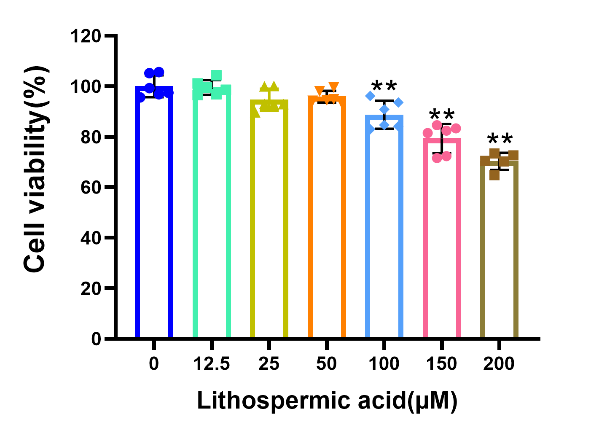 | 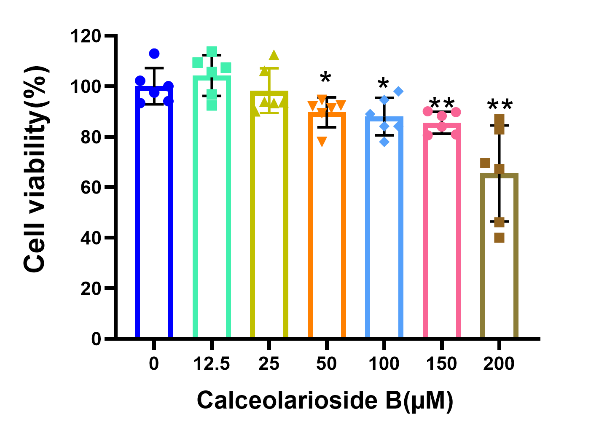 |
| --- | --- | --- |

| 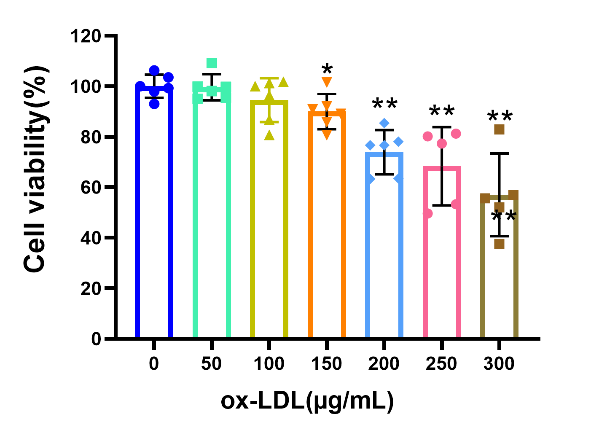 | 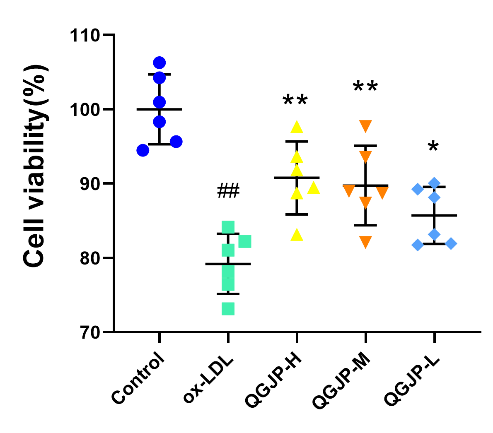 |
| --- | --- |
| Fig.S1 Viability of HUVECs treated with ox-LDL and QGJP (x̄±s, n=6).  ^##^*p*＜0.01 compared with Control group; ^#^*p*＜0.05 compared with Control group;^**^*p*＜0.01 compared with Model group; ^*^*p*＜0.05 compared with Model group.  **S2. The effect of SAA, LA, and CAB on THP-1 cell viability.** | |

Fig.S2. Viability of THP-1 cells treated with SAA, LA, and CAB (x̄±s, n=6).

^##^*p*＜0.01 compared with Control group; ^#^*p*＜0.05 compared with Control group;^**^*p*＜0.01 compared with Model group; ^*^*p*＜0.05 compared with Model group.

**S3. Effect of QGJP on MCT4 mRNA expression levels in THP-1 cells.**

**Materials and methods**

**RNA extraction and RT-qPCR**

Following the manufacturer^,^s protocol, RNA was isolated from cultivated cells using RNAsimple Total RNA Kit (TIANGEN, China). The cDNA synthesis was then performed using PrimeScript^TM^ RT reagent Kit (Takara, China). Gene expression was then measured by qPCR using TB Green® Premix Ex Taq^TM^ (Takara, China) on an LightCycler 96 real-time PCR system (Roche, USA) and the mRNA expression was normalized to GAPDH as a reference. Primer sequences are listed in Table S1.

Table S1: List of primers sequences used in the study

| Gene Name | Primer | |
| --- | --- | --- |
|  | Forward sequence | Reverse sequence |
| MCT4 | CCATGCTCTACGGGACAGG | GCTTGCTGAAGTAGCGGTT |
| GAPDH | CACCATTGGCAATGAGCGGTTC | GGCTGTTGTCATACTTCTCATGG |

**Results:**


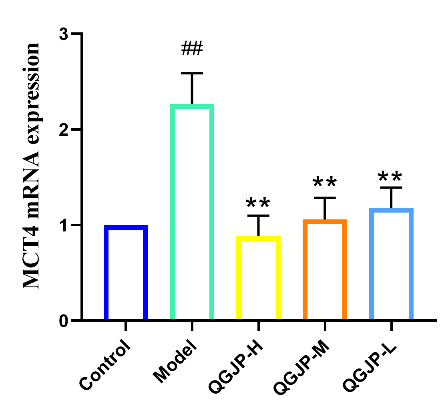


Fig.S3. MCT4 mRNA expression in QGJP-treated THP-1 cells.

^##^*p*＜0.01 compared with Control group; ^#^*p*＜0.05 compared with Control group;^**^*p*＜0.01 compared with Model group; ^*^*p*＜0.05 compared with Model group.

**S4. Transcriptomic Analysis of the Aorta in AS Mice**

**Materials and methods**

Total RNA was isolated and purified using TRIzol (Thermo Fisher, 15596018) according to the manufacturer’s protocol. The quantity and purity of total RNA were assessed using a NanoDrop ND-1000 spectrophotometer (NanoDrop, Wilmington, DE, USA), and RNA integrity was evaluated using a Bioanalyzer 2100 (Agilent, CA, USA). Samples with a concentration >50 ng/μL, RIN value >7.0, and total RNA >1 μg were deemed suitable for downstream experiments. Polyadenylated (polyA) mRNA was selectively enriched through two rounds of purification using oligo(dT) magnetic beads (Dynabeads Oligo (dT), cat. 25-61005, Thermo Fisher, USA). The captured mRNA was fragmented under elevated temperature using a magnesium ion-based fragmentation kit (NEBNext® Magnesium RNA Fragmentation Module, cat. E6150S, USA) at 94 °C for 5–7 minutes. Fragmented RNA was reverse transcribed into cDNA using SuperScript™ II Reverse Transcriptase (Invitrogen, cat. 1896649, CA, USA). Second-strand synthesis was subsequently performed using E. coli DNA polymerase I (NEB, cat. M0209, USA) and RNase H (NEB, cat. M0297, USA), converting the RNA-DNA hybrids into double-stranded DNA (dsDNA), during which dUTP Solution (Thermo Fisher, cat. R0133, CA, USA) was incorporated into the second strand. The resulting dsDNA was end-repaired to generate blunt ends, followed by the addition of an adenine (A) base to the 3′ ends to facilitate ligation with adapters featuring a complementary thymine (T) overhang. Fragment size selection and purification were performed using magnetic beads. The second strand was digested with UDG enzyme (NEB, cat. M0280, MA, USA), and the library was amplified by PCR under the following conditions: initial denaturation at 95 °C for 3 minutes; 8 cycles of denaturation at 98 °C for 15 seconds, annealing at 60 °C for 15 seconds, and extension at 72 °C for 30 seconds; followed by a final extension at 72 °C for 5 minutes. This generated a strand-specific cDNA library with an insert size of approximately 300 bp ± 50 bp. Finally, paired-end sequencing (PE150) was performed on the Illumina NovaSeq™ 6000 platform following the manufacturer’s standard protocol. Following the acquisition of raw sequencing data, quality filtering was first performed to obtain high-quality clean data. The clean reads were then aligned to the reference genome of the species under investigation, followed by analyses including gene expression quantification, differential gene expression analysis, and enrichment analysis.

**Results:**

| 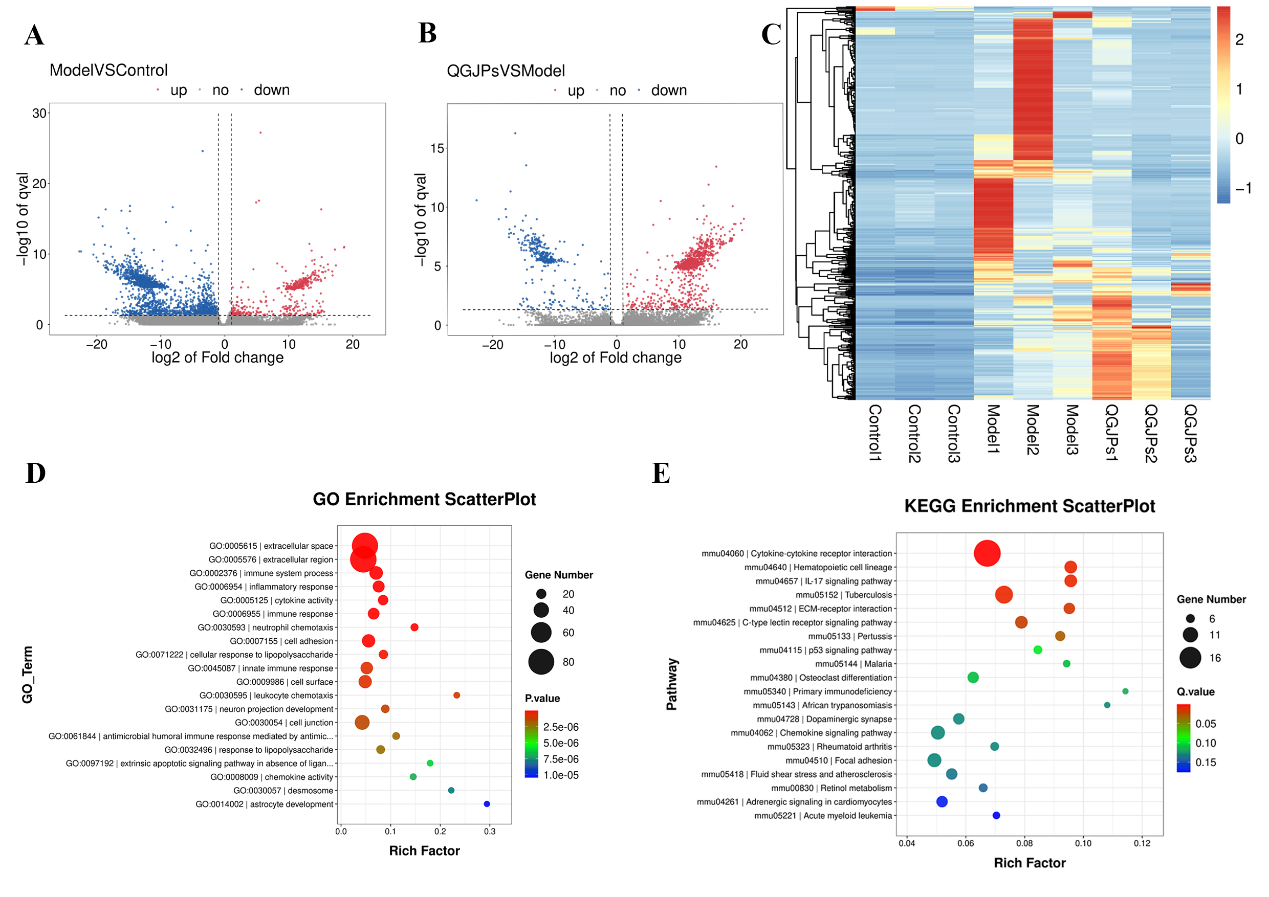 |
| --- |
| Fig.S4. AS mouse aortic tissue transcriptomics analysis. (A) Volcano plot of gene expression differences in Model group vs Control group. (B) Volcano plot of gene expression differences in QGJP group vs Model group. (C) Cluster heatmap of differentially expressed genes (DEGs) among the control groups, model groups, and QGJP-H groups. (D, E) Enrichment analysis of Gene Ontology (GO) biological processes and KEGG pathways for the upregulated and downregulated differentially expressed genes (DEGs) (adj. *p＜*0.05, TOP20 terms shown). The significance of enrichment is indicated by color hue, and the number of DEGs within each term is represented by bubble size (n=3). |

**S4. Analysis of components of QGJP by UHPLC-HRMS**

Table S2 Analysis of components of QGJP by UHPLC-HRMS

| Number | m/z | compound name | adduct | score | Herb source |
| --- | --- | --- | --- | --- | --- |
| 1 | 285.0761 | Genkwanin | [M+H]+ | 0.9919 | *Artemisia capillaris* Thunb |
| 2 | 295.1331 | Tanshinone IIA | [M+H]+ | 0.9881 | *Salvia miltiorrhiza* Bunge |
| 3 | 153.0543 | 2-Hydroxy-4-methoxybenzaldehyde | [M+H]+ | 0.8056 | *Bupleurum chinense* DC |
| 4 | 157.0135 | Citric acid | [M+H-2H2O]+ | 0.9005 | *Rheum palmatum L Polygonum cuspidatum Sieb.et Zucc. Crataegus pinnatifida Bunge* |
| 5 | 179.0342 | 5,7-Dihydroxychromone | [M+H]+ | 0.9969 | *Senna tora (L.) Roxb* |
| 6 | 188.0704 | Tryptophan | [M+H-NH3]+ | 0.9789 | *Bupleurum chinense DC* |
| 7 | 229.0862 | Resveratrol | [M+H]+ | 0.9978 | *Polygonum cuspidatum Sieb.et Zucc.* |
| 8 | 233.0812 | 7-Acetyl-3,8-dihydroxy-6-methylnaphthalen-1-yl .beta.-D-glucopyranoside | [M+H-C6H10O5]+ | 0.9358 | *Rheum palmatum L* |
| 9 | 257.0423 | Purpurin | [M+H]+ | 0.9862 | *Senna tora (L.) Roxb* |
| 10 | 271.0604 | Baicalein | [M+H]+ | 0.9997 | *Plantago asiatica L* |
| 11 | 271.0606 | Apigenin | [M+H]+ | 0.9994 | *Salvia miltiorrhiza Bunge Polygonum cuspidatum Sieb.et Zucc.* |
| 12 | 277.0862 | Tanshinone A | [M+H]+ | 0.9438 | *Salvia miltiorrhiza Bunge* |
| 13 | 287.0553 | Luteolin | [M+H]+ | 0.9997 | *Salvia miltiorrhiza Bunge Polygonum cuspidatum Sieb.et Zucc. Plantago asiatica L* |
| 14 | 303.1229 | Isomucronulatol | [M+H]+ | 0.9942 | *Astragalus membranaceus (Fisch.) Bunge* |
| 15 | 315.0866 | Cirsimaritin | [M+H]+ | 0.9615 | *Artemisia capillaris Thunb* |
| 16 | 341.066 | Lithospermic acid | [M+H-C9H10O5]+ | 0.9503 | *Salvia miltiorrhiza Bunge* |
| 17 | 345.0973 | Eupatrin | [M+H]+ | 0.9513 | *Artemisia capillaris Thunb* |
| 18 | 369.116 | Aucubin | [M+Na]+ | 0.9997 | *Plantago asiatica L* |
| 19 | 439.1006 | Chrysophanol 8-O-beta-D-glucoside | [M+Na]+ | 0.9907 | *Senna tora (L.) Roxb Rheum palmatum L* |
| 20 | 447.1292 | Calycosin-7-o-beta-d-glucoside | [M+H]+ | 0.9996 | *Astragalus membranaceus (Fisch.) Bunge* |
| 21 | 461.1086 | Oroxindin | [M+H]+ | 0.9998 | *Bupleurum chinense DC* |
| 22 | 463.0985 | 6-{[2-(3,4-dihydroxyphenyl)-5-hydroxy-4-oxo-4H-chromen-7-yl]oxy}-3,4,5-trihydroxyoxane-2-carboxylic acid | [M+H]+ | 0.9954 | *Polygonum cuspidatum Sieb.et Zucc.* |
| 23 | 463.1241 | Hispiduloside | [M+H]+ | 0.9976 | *Plantago asiatica L* |
| 24 | 137.0235 | 4-Hydroxybenzoic acid | [M-H]- | 0.9988 | *Crataegus pinnatifida Bunge* |
| 25 | 153.0187 | Gentisic acid | [M-H]- | 0.9956 | *Plantago asiatica L* |
| 26 | 285.0404 | Citreorosein | [M-H]- | 0.9976 | *Polygonum cuspidatum Sieb.et Zucc.* |
| 27 | 285.0405 | Luteolin | [M-H]- | 0.9992 | *Salvia miltiorrhiza Bunge Polygonum cuspidatum Sieb.et Zucc. Plantago asiatica L* |
| 28 | 285.0405 | Scutellarein | [M-H]- | 0.7192 | *Plantago asiatica L* |
| 29 | 299.0561 | Fallacinol | [M-H]- | 0.9513 | *Polygonum cuspidatum Sieb.et Zucc.* |
| 30 | 315.0512 | Isorhamnetol | [M-H]- | 0.9927 | *Bupleurum chinense DC Crataegus pinnatifida Bunge Artemisia capillaris Thunb Astragalus membranaceus (Fisch.) Bunge'}* |
| 31 | 328.0586 | Eupatrin | [M-H-CH3]- | 0.8395 | *Artemisia capillaris Thunb* |
| 32 | 405.1192 | Astringin | [M-H]- | 0.9989 | *Polygonum cuspidatum Sieb.et Zucc.* |
| 33 | 433.0778 | Reynoutrin | [M-H]- | 0.961 | *Polygonum cuspidatum Sieb.et Zucc.* |
| 34 | 435.1297 | Polydatin | [M+HCOO]- | 0.997 | *Polygonum cuspidatum Sieb.et Zucc.* |
| 35 | 445.0782 | Baicalin | [M-H]- | 0.9955 | *Bupleurum chinense DC Plantago asiatica L Salvia miltiorrhiza Bunge* |
| 36 | 449.1456 | Deoxyrhapontin | [M+HCOO]- | 0.9695 | *Rheum palmatum L* |
| 37 | 455.3533 | Betulinic acid | [M-H]- | 0.9996 | *Astragalus membranaceus (Fisch.) Bunge'}* |
| 38 | 461.0673 | 6-{[2-(3,4-dihydroxyphenyl)-5-hydroxy-4-oxo-4H-chromen-7-yl]oxy}-3,4,5-trihydroxyoxane-2-carboxylic acid | [M-H]- | 0.9976 | *Polygonum cuspidatum Sieb.et Zucc.* |
| 39 | 461.0732 | Luteolin 7-glucuronide | [M-H]- | 0.9983 | *Polygonum cuspidatum Sieb.et Zucc.* |
| 40 | 463.0886 | Hirsutrin | [M-H]- | 0.982 | *Bupleurum chinense DC Artemisia capillaris Thunb  Polygonum cuspidatum Sieb.et Zucc.  Astragalus membranaceus (Fisch.) Bunge* |
| 41 | 465.1404 | Rhapontin | [M+HCOO]- | 0.9458 | *Rheum palmatum L* |
| 42 | 475.1248 | Ononin | [M+HCO2]- | 0.9998 | *Astragalus membranaceus (Fisch.) Bunge* |
| 43 | 477.1403 | Calceolarioside B | [M-H]- | 0.9954 | *Plantago asiatica L* |
| 44 | 485.3275 | Quillaic acid | [M-H]- | 0.9998 | *Polygonum cuspidatum Sieb.et Zucc.* |
| 45 | 491.1196 | Calycosin-7-o-beta-d-glucoside | [M+HCO2]- | 0.9579 | *Astragalus membranaceus (Fisch.) Bunge* |
| 46 | 493.1141 | Salvianolic acid A | [M-H]- | 0.9939 | *Salvia miltiorrhiza Bunge* |
| 47 | 609.1465 | Rutin | [M-H]- | 0.9833 | *Bupleurum chinense DC. Crataegus pinnatifida Bunge Artemisia capillaris Thunb Astragalus membranaceus (Fisch.) Bunge* |
